# Comparing Metal–Halide and −Oxygen Adducts in Oxidative C/O–H Activation: Au^III^–Cl versus Au^III^–OH

**DOI:** 10.1021/acs.inorgchem.1c02222

**Published:** 2021-09-28

**Authors:** Marta Lovisari, Robert Gericke, Brendan Twamley, Aidan R. McDonald

**Affiliations:** School of Chemistry, Trinity College Dublin, The University of Dublin, College Green, Dublin 2, Ireland

## Abstract

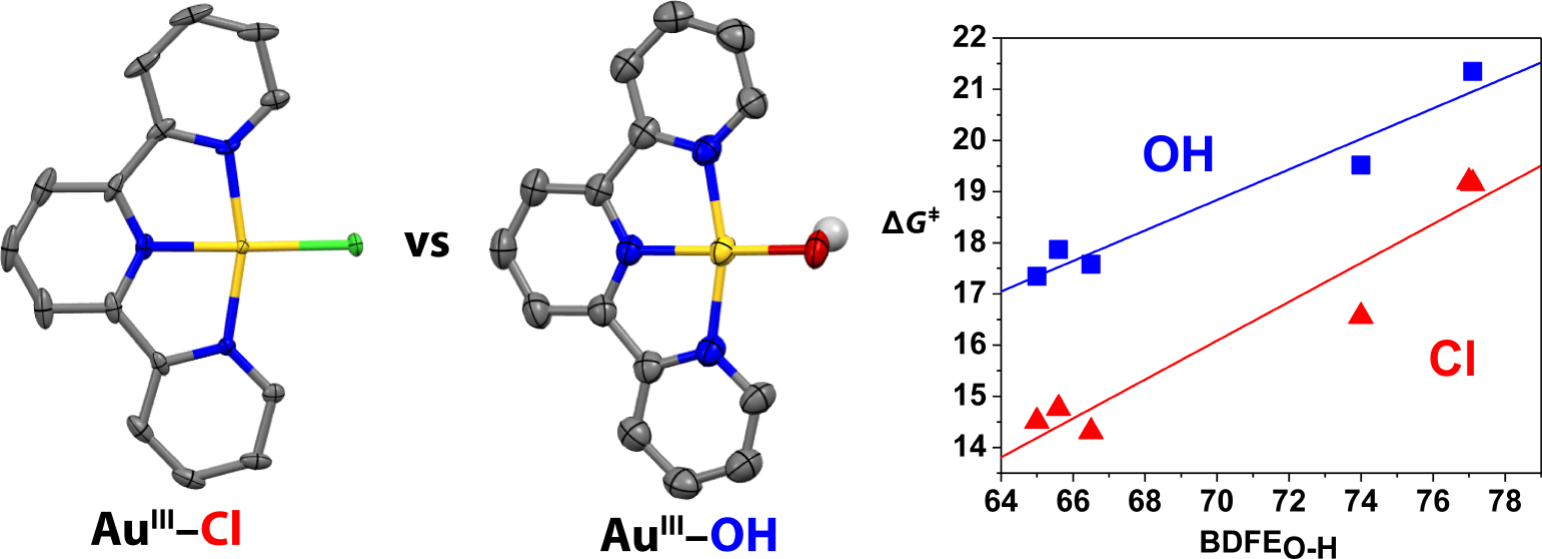

High-valent metal–halides have come to prominence as highly effective oxidants. A direct comparison of their efficacy against that of traditional metal–oxygen adducts is needed. [Au^III^(Cl)(terpy)](ClO_4_)_2_ (**1**; terpy = 2,2′:6′,2-terpyridine) readily oxidized substrates bearing O–H and C–H bonds via a hydrogen atom transfer mechanism. A direct comparison with [Au^III^(OH)(terpy)](ClO_4_)_2_ (**2**) showed that **1** was a kinetically superior oxidant with respect to **2** for all substrates tested. We ascribe this to the greater thermodynamic driving force imbued by the Cl ligand versus the OH ligand.

## Abstract 2

We report a direct comparison of the efficacy of metal−halide oxidants to those of traditional metal−oxygen adducts. Au^III^−Cl and Au^III^−OH oxidants, supported by the same ancillary ligand, readily oxidized substrates via a hydrogen atom transfer mechanism. The Au^III^−Cl oxidant was kinetically superior for all substrates. We ascribed this to the greater thermodynamic driving force imbued by the Cl ligand versus the OH ligand.

## Introduction

The oxidative functionalization of inert saturated hydrocarbons remains a challenge.^1,2^ High-valent metal oxidants are ideal candidates for C–H bond activation via a hydrogen atom transfer (HAT) mechanism. High-valent metal–halides have attracted attention as acting as potent oxidants.^3−6^ We showed that a Ni^III^–Cl entity performs HAT and postulated that the thermodynamic driving force for HAT was the bond dissociation free energy (BDFE) of the free HCl product.^3^ Subsequently, a Ni^III^–F complex showed 4300-fold enhanced rate constants in the oxidation of xanthene and CHD compared to those of Ni^III^–Cl, the enhanced reactivity attributed to a stronger H–F bond.^4^ An analogous Cu^III^–F was recently found to be capable of performing oxidative fluorination using similar HAT mechanisms.^5^ Doyle and co-workers have pioneered the application of Ni^II^–Cl catalysts, under photolysis conditions, yielding oxidatively cross-coupled sp^3^ C–H bonds. They postulated that an intermediary Ni^III^–Cl moiety released Cl^•^, which was the species responsible for HAT oxidation of substrates.^7−10^ This is in contrast with the mechanistic hypothesis of the high-valent metal–halide species performing the HAT step. The discovery of the capability of high-valent metal–halides in hydrocarbon oxidation leads us to assess the differences between traditional high-valent metal–oxygen oxidants and high-valent metal–halides.

Au–oxygen adducts have been proposed as active oxidants in both heterogeneous and homogeneous oxidation catalysis.^11−16^ Interestingly, Shilov reported hydrocarbon oxidation promoted by NaAuCl_4_ and Au(PPh_3_)Cl in the presence of hydrogen peroxide.^11^ An elusive terminal Au^III^=O group was the postulated oxidant,^11^ although discrete Au^III^–Cl species could be effective oxidants. We recently reported the oxidative reactivity of [Au^III^(OH)(terpy)](ClO_4_)_2_ (**2**; terpy = 2,2′:6′,2-terpyridine) toward substrates bearing C–H and O–H bonds that we determined to follow a HAT mechanism.^16^ A comparable Au^III^–Cl complex, [Au^III^(Cl)(terpy)](ClO_4_)_2_ (**1**), allows us to perform a direct comparison of oxidants bearing an oxygen ligand versus those bearing halide ligands and to explore the efficacy of Au–Cl oxidants. Herein, we explore both Au complexes under the exact same experimental conditions, allowing us to consider whether metal–halides or −hydroxides are preferable oxidants for hydrocarbon oxidation.

## Results and Discussion

**Scheme 1:**
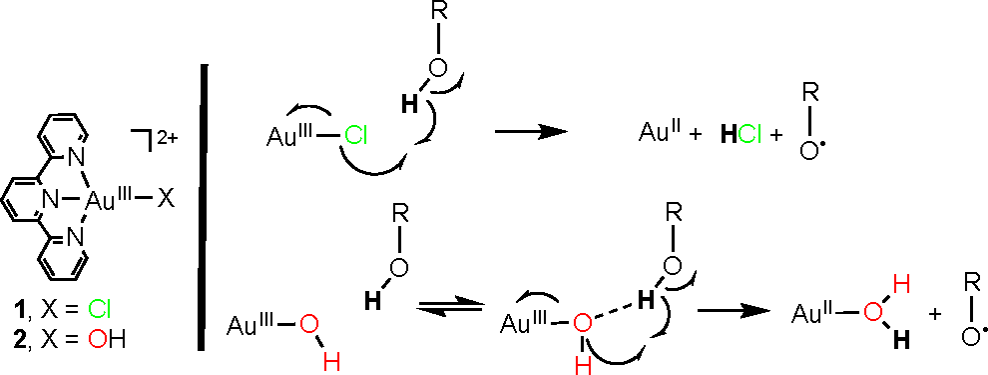
Structures of **1** and **2** and Mechanisms of HAT Oxidation

**Figure 1:**
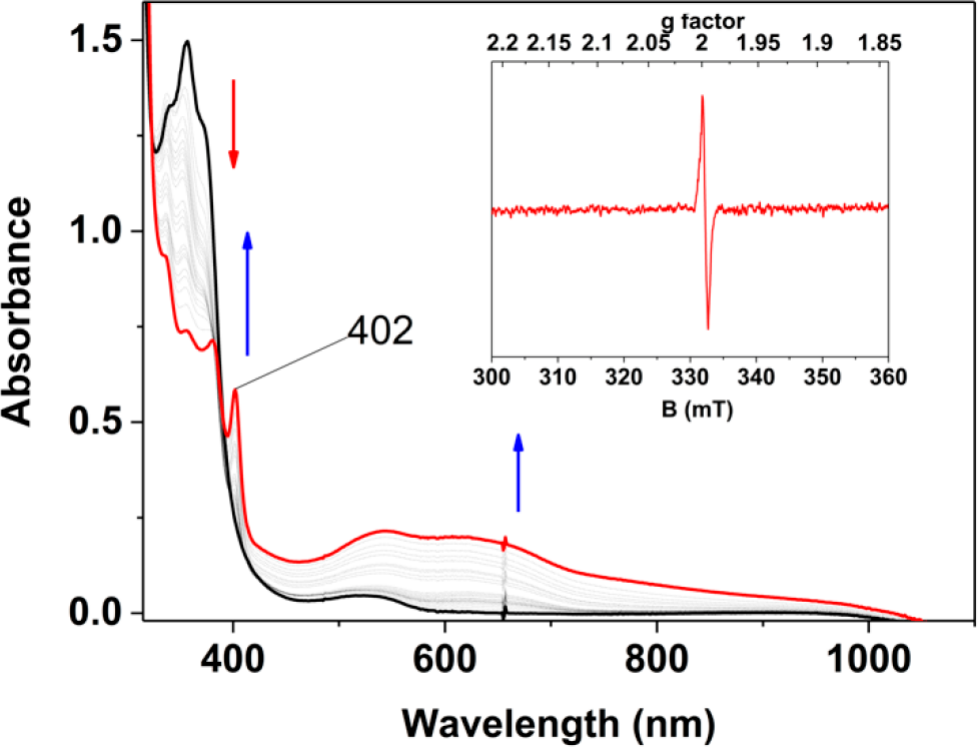
Electronic absorption spectra of the reaction of **1** (DMF, 25 °C, 0.20 mM) with 2,4,6-TTBP (600 equiv): black trace: t = 0 s, red trace t = 2000 s. The inset shows the X-band EPR spectrum of the reaction mixture at 2000 s. The EPR spectrum was acquired from a frozen DMF solution and measured at 77 K with a 0.2 mW microwave power and a 0.2 mT modulation amplitude.

**1** was synthesized as reported (Scheme 1, Figures S1 and S2, and Tables S1 and S2).^17^ The electronic absorption spectrum of **1** in *N*,*N*-dimethylformamide (DMF) showed bands at 375 and 357 nm and a shoulder at 338 nm (Figure 1 and Figures S3–S5). These features are typical of complexes supported by substituted terpyridine ligands and can be assigned to the intraligand transitions perturbed by the complexation to a metal center.^18^ A feature at 530 nm has been attributed to LMCT (ligand-to-metal charge transfer) and ILCT (intraligand charge transfer) contributions.^18−20^ Cyclic voltammetry on **1** showed an irreversible reduction peak at *E*_red_ = 0.18 V versus the ferrocene/ferrocenium (Fc/Fc^+^) standard and an irreversible oxidation wave at high potential [*E*_ox_ = 0.74 V (Figure S6)]. The 0.18 V peak was assigned to a reduction either of the starting Au^III^ complex or of a species formed upon the oxidation event. This reduction event falls at a potential higher than that of the one observed for **2** (−0.13 V).^16,21,22^

To perform a direct comparison of metal–halide **1** and metal–hydroxide **2**, we explored their reactivity toward substrates containing weak O–H or C–H bonds, under the same conditions. When **1** (0.15 mM, 25 °C, DMF) was reacted with 2,4,6-tris-*tert*-butylphenol (2,4,6-TTBP, >150 equiv), the electronic absorption spectrum showed first the formation of a band at 402 nm (maximum yield at 2000 s) and the decay of the bands at 357 and 375 nm (Figure 1). In a second phase of the reaction, a slower growth of features between 500 and 1000 nm over 3000 s was observed. A dark purple solution was observed at the end of the reaction, which we attribute to the formation of colloidal gold, in analogy to what was previously observed for **2**.^16^

The feature at 402 nm was assigned to the 2,4,6-tris-*tert*-butylphenoxyl radical, which we have previously independently synthesized.^16^ The yield of this radical species was determined to be 80 ± 12% by ultraviolet–visible (UV–vis) spectroscopy (Table S3). It must be noted that this yield was determined with respect to the concentration of **1** if each molecule of **1** was responsible for one H atom transfer *in the initial phase of the reaction* (0 - to - 2000 s). Given the observation of colloidal gold at the end of the reaction (3000 s), it is possible that one, two, or three electrons and protons might be theoretically transferred to each equivalent of **1**, which could lower the estimated yield of the phenoxyl radical with respect to the concentration of **1**. We have not considered the decay product (presumed to be Au^II^OH_2_) as an active oxidant in our calculations of yields because our focus was directed toward the first step of the reaction, where the stoichiometry was assumed to be 1:1. The assumption described above was made for all of the product yields reported in this work and in our previous work,^16^ allowing a comparison of the yields of the products between complexes **1** and **2**. An X-band electron paramagnetic resonance (EPR) spectrum taken after 2000 s (maximum yield of 402 nm) showed a signal at *g* = 2.00 (Figure 1). This spectrum displayed the same EPR *g* value of the independently synthesized 2,4,6-tris-*tert*-butylphenoxyl radical, confirming the identity of the product. The yield of the radical was determined to be 75 ± 20% by EPR (Table S3) with respect to the concentration of **1**. We conclude that the radical was formed as a result of a formal one-proton, one-electron oxidation of 2,4,6-TTBP by **1**.

The reaction kinetics were followed by monitoring the decay at 375 nm over time in the initial phase of the reaction (0 - to - 2000 s). Pseudo-first-order rate constants (*k*_obs_) were obtained by exponential fitting of the plot of absorbance versus time. The plot of *k*_obs_ versus 2,4,6-TTBP concentration exhibited a linear correlation (Figure S7), and from the slope, the second-order reaction rate constant, *k*_2_, was determined to be 0.056 M^–1^ s^–1^ (Table S4). The final product (after Au particle formation and work-up) was found to be 2,6-di-*tert*-butylquinone (2,6-DTBQ), and its yield was estimated by GC to be 71 ± 6%, considering that for every Au in **1** a two-electron reaction occurred (2,6-DTBQ is a two-electron, two-proton oxidation product of 2,4,6-TTBP).^23^ The other product of this reaction could either be *tert*-butanol or isobutylene, but we could not identify these products by ^1^H NMR or GC-FID. **1** was thus capable of proton-coupled electron transfer (PCET) oxidative activation of phenolic O–H bonds.

To gain mechanistic insight, **1** was reacted with a family of substrates bearing O–H bonds: 4-methoxy-2,6-di-*tert*-butylphenol (4-CH_3_O-2,6-DTBP), 4-methyl-2,6-di-*tert*-butylphenol (4-CH_3_-2,6-DTBP), and 4-X-1-hydroxy-2,2,6,6-tetramethylpiperidines (4-X-TEMPOH, where X = H, O, or CH_3_O). For all substrates, the electronic absorption spectra displayed the decay of the complex features at 357 and 375 nm to obtain *k*_obs_ and *k*_2_ values as described above (Table S4 and Figures S8–S17). X-Band EPR measurements of the reaction mixtures for 4-X-TEMPOH substrates gave a rhombic signal at *g*_av_ = 2.00, typical of 4-X-TEMPO^•^ species (Figures S18 and S19).^24^ The radical products were obtained in ∼80% yields (Table S3). When **1** was reacted with 4-CH_3_O-2,6-DTBP, no electronic absorption or EPR signals indicating accumulation of a radical species were observed (Figure S9). This suggests that the 4-methoxy-2,6-di-*ter*t-butylphenoxyl radical likely decayed too quickly to be observed. ^1^H NMR analysis of this reaction showed 2,6-DTBQ (yield of 96 ± 7% by GC-FID)^16^ and C*H*_3_OH had formed (Figure S10).^16^ These compounds are likely formed by PCET oxidation of 4-CH_3_O-2,6-DTBP by **1**. Overall, substrates bearing weak O–H bonds reacted initially with **1** to yield stable O-based radical species that indicate a PCET oxidation by **1**.

For the reaction of **1** with deuterated (OD) 4-CH_3_O-2,6-DTBP, a *k*_2_ of 2.34 M^–1^ s^–1^ was determined, yielding a primary kinetic isotope effect (KIE) of 1.9 (Table S4 and Figure S11). This indicates that proton or H atom transfer was involved in the rate-limiting step. The KIE is in the range typical of high-valent metal–halides and M–OX PCET oxidants.^3,24−28^

**Figure 2:**
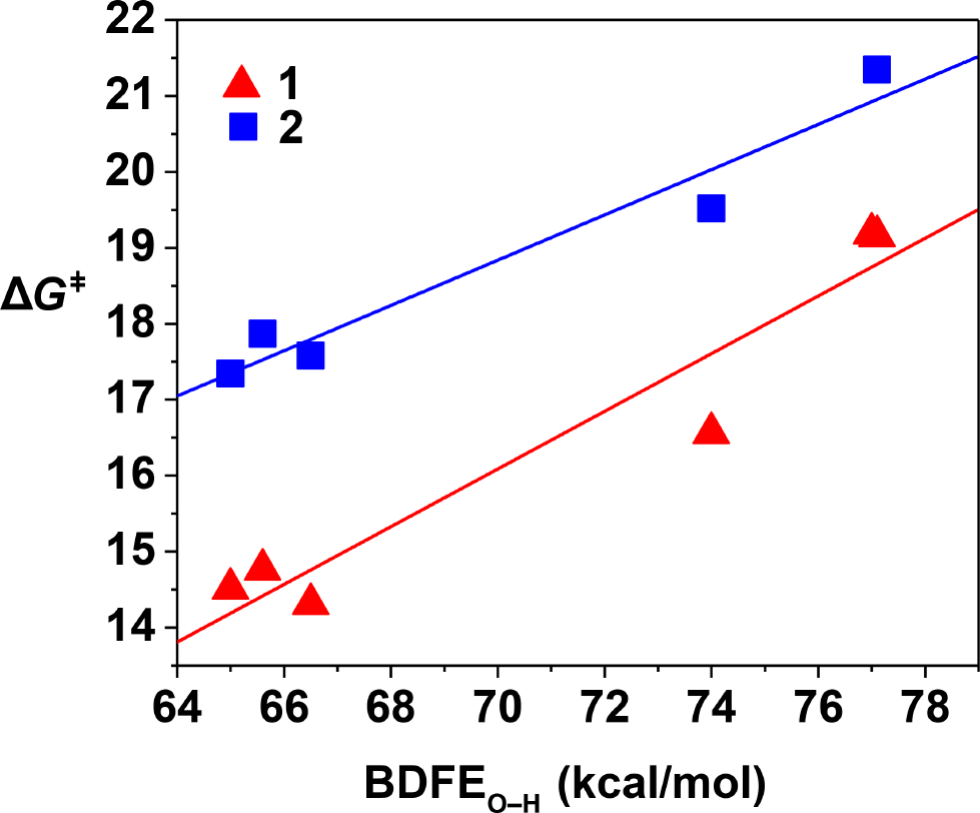
Plot of Δ*G*^⧧^ vs BDFE_O–H_ of the substrates for **1** (red triangles) and **2** (blue squares).^16^ The values of Δ*G*^⧧^ were determined from *k*_2_ via the Eyring equation (for **1**, slope = 0.39, Pearson’s *r* = 0.9637, and adjusted *R*^2^ = 0.9109; for **2**, slope = 0.30, Pearson’s *r* = 0.9745, and adjusted *R*^2^ = 0.9328). BDFE_O–H_ values are not available for these substrates in DMF; hence, we have plotted BDFE_O–H_ values for CH_3_CN.^29^

The reactions with 4-X-TEMPOH substrates showed relatively fast rates, whereas those with phenols showed relatively slower rates. The extent of the variation in *k*_2_ can be rationalized by plotting Gibbs free energies of activation (Δ*G*^⧧^, derived from *k*_2_) against BDFE_O–H_ [Bell–Evans–Polanyi plot (Figure 2)]. A linear fit with a slope of 0.39 was obtained, which is close to the ideal value (0.5) for a concerted proton and electron transfer (CPET) or hydrogen atom transfer (HAT) mechanism as predicted by Marcus theory.^29−32^ This value falls in the range of Δ*G*^⧧^/Δ(BDFE) slopes ascribed to HAT mechanisms for transition metal-based oxidants (0.15–0.7)^30,33^ and is also close to the value of 0.30 reported for **2** (Figure 2).^16^

Activation energy parameters were determined via an Arrhenius analysis for the reaction of **1** with 4-CH_3_O-2,6-DTBP (Figure S20). The activation enthalpy (Δ*H*^⧧^) was determined to be 12.5 kcal mol^–1^, and the activation entropy (Δ*S*^⧧^) was determined to be −18 cal mol^–1^ K^–1^. The large, negative Δ*S*^⧧^ is consistent with a metal-based oxidant: during a HAT reaction, the vibrational entropy contribution is much larger for metal-mediated HAT compared to reactions involving organic radicals.^34,35^ The Δ*H*^⧧^ for **2** was 2.6 kcal/mol lower than that measured for **1**, a small difference that we do not expect to have a marked impact on the final Δ*G*^⧧^. However, the value of Δ*S*^⧧^ for **1** was markedly smaller than for **2** (Table 1). The activation parameters are consistent with a metal-mediated HAT oxidation reaction by **1**, with parameters indicating that free Cl atom-mediated HAT oxidation of substrate (expected Δ*S*^⧧^ < 5 cal mol^–1^ K^–1^) is unlikely.

**Table 1:** Thermodynamic and Kinetic Parameters for the Reactions of **1** and **2** with Substrates Bearing O–H Bonds

complex	Δ*H*^⧧^ (kcal/mol)	Δ*S*^⧧^ (cal mol^–1^ K^–1^)	Δ*G*^⧧^ (kcal/mol)^a^,^b^	Δ*G*^⧧^ (kcal/mol)^a^,^c^	Δ*G*^⧧^/Δ(BDFE)	KIE^a^
**1**	12.5	–18	17.8	16.5	0.39	1.9
**2**	9.9	–33	19.7	19.5	0.30	2.9

a Values calculated from the reaction with 4-CH_3_O-2,6-DTBP.

b From an Eyring plot.

c From a Bell-Evans-Polanyi plot.

The Δ*G*^⧧^ for the reaction of both complexes with 4-CH_3_O-2,6-DTBP was calculated from both the Arrhenius analysis (Figure S20) and the Bell–Evans–Polanyi plot (Figure 2). For **1**, values of 17.8 and 16.5 kcal/mol, respectively, were found (Table 1), whereas for **2**, values of 19.7 and 19.5 kcal/mol, respectively, were obtained. Thus, with both methods, **1** showed a lower Δ*G*^⧧^ for the HAT oxidation of a substrate compared to that of **2** (Scheme 1). Overall, **1** was consistently a kinetically more competent oxidant that **2** (Figure 2), with the activation barrier on average 2–3 kcal/mol lower for all substrates explored.

It is important to note that the profiles of the kinetics of reaction for both complexes were different. **1** showed linear kinetics even at high substrate concentrations, whereas **2** showed saturation kinetics. For **2**, we attributed the saturation behavior to a pre-equilibrium phase prior to the HAT step, where a substrate was hydrogen bonding to the hydroxide ligand prior to HAT.^16,24^ For **1**, the presence of such an intermediate does not occur, presumably because the Cl ligand in **1** is less prone to forming hydrogen bonds.^36^ This phenomenon was observed for a pair of Mn^III^–OH and Mn^III^–Cl species, where a higher reorganization energy was observed for Mn^III^–OH due to its tendency to form a hydrogen bonding network.^37^

### Understanding the Differences in Reactivity

To examine the driving force responsible for the higher competency of **1** with respect to **2**, for HAT oxidants the thermodynamic parameter to explore is the BDFE_H–Cl/O_ of the reduced conjugate acids obtained after HAT {i.e., [Au^II^(···HCl)(terpy)]^2+^ for **1** and [Au^II^(H_2_O)(terpy)]^2+^ for **2**}.^30^ Due to the tendency of Au^II^ to undergo disproportionation to yield colloidal gold, the synthesis of these complexes was not possible.^15,16,22,38,39^ We observed no evidence for Au(II) in any of our EPR analyses of the reaction mixtures. We could estimate BDFE values for both complexes using the Bordwell equation: BDFE_H–X_ = 1.37 (p*K*_a_) + 23.06 (*E*_1/2_^II/III^) + *C*_G_.^29^ To do this, we attempted to determine p*K*_a_ and redox potential values for the Au^III^ H–X adducts of **1** and **2** {[Au^III^(···HCl)(terpy)]^3+^ (**1H**^**+**^) and [Au^III^(H_2_O)(terpy)]^3+^ (**2H**^**+**^)}, which would allow us to estimate BDFE_H–X_ values in [Au^II^(···HCl)(terpy)]^2+^ and [Au^II^(H_2_O)(terpy)]^2+^.

To understand the nature of the [Au^II^(···HCl)(terpy)]^2+^ product, we reacted **1** with carboxylic acids observing very small changes in the ultraviolet region of the electronic absorption spectra (Figures S21 and S22 and Table S5). We were unable to isolate the product of this reaction. ^1^H NMR analysis of the reaction between **1** and trifluoroacetic acid showed the appearance of new resonances in the terpyridine region [8.0–9.5 ppm (Figures S23 and S24)] together with a marked shift and broadening of the water residual peak. We reacted free terpyridine with trifluoroacetic acid, which yielded a new set of resonances for protonated terpy that were markedly different from those obtained when trifluoroacetic acid was added to **1** (Figure S25). We concluded that upon addition of an acid to **1**, protonated terpy was not formed, suggesting that other sites might be more likely protonated. We therefore postulated that if the ancillary Cl^–^ is protonated, HCl could be formed and released, even though we do not have experimental proof for this hypothesis due to the instability of this adduct. We postulated that free HCl could act as the thermodynamic driver for HAT by **1**. To the best of our knowledge, the isolation of a metal–HCl coordination compound is unprecedented.

In an effort to measure the p*K*_a_ of [Au^III^(H_2_O)(terpy)]^3+^ (**2H**^**+**^), we reacted H^+^ donors with **2** (Figures S26–S28 and Table S6). A blue-shift of the bands in the UV and visible regions was observed to yield a new species defined as **2H**^**+**^. The shift and the broadening of the water residual peak in polar aprotic solvents have been ascribed to the increase in the water content in the reaction vessel, which perturbed the network of hydrogen bonds yielding the shift and the broadening of the peak.^40,41^ The formation of this species was reversible through addition of 2,6-lutidine (Figure S29). As determined by ^1^H NMR, addition of pyridinium triflate (PyHOTf, 5 equiv) to **2** resulted in the disappearance of the resonance at δ = 6.35 ppm, assigned to the hydroxide ligand. We subsequently added 2,6-lutidine and observed the reappearance of the resonance at δ = 6.35 ppm, indicating that the hydroxide ligand was restored (Figure S26). We concluded this acid/base reaction was consistent with the interconversion of [Au^III^(OH)(terpy)]^2+^ (**2**) and [Au^III^(H_2_O)(terpy)]^3+^ (**2H**^**+**^), although it is also possible that the H_2_O ligand is not coordinated to the Au^III^ ion. We were unable to prepare this species on a synthetic scale, and therefore, we have assumed **2H**^**+**^ has a [Au^III^(H_2_O)(terpy)]^3+^ structure.

We proceeded to measure the equilibrium constant (*K*_a_) for the reaction **2** + PyHOTf → **2H**^**+**^ + Py to determine the p*K*_a_ for **2H**^**+**^ (see the Supporting Information for details). The plot of [**2H**^**+**^][Py]/[**2**] against [PyHOTf] showed a linear trend considering the absorbance data taken at 369 nm (Figure S30). The slope of this plot is equal to the equilibrium constant for the protonation of **2** (*K*_eq_), and a final p*K*_a_ of **2H**^**+**^ was calculated to be 4.54. Cyclic voltammetry measurements on **2H**^**+**^ showed the presence of a reversible redox wave at *E*_1/2_ = 0.22 V versus Fc/Fc^+^ (Figures S32 and S33). We assigned this redox wave to the reversible reduction/oxidation of **2H**^**+**^, which in contrast to **2** (*E*_red_ = −0.13 V) demonstrated a reversible redox event. Considering the Bordwell equation, we could estimate a BDFE_O–H_ of 81 ± 3 kcal/mol for the aquo ligand in [Au^II^(H_2_O)(terpy)]^2+^. This result is consistent with the range (and limits) of the BDFE_O–H_ of the substrates that reacted with **2** (65–82 kcal/mol).^16,29^

The differences in reactivity between **1** and **2** can thus be compared with the differences in BDFE_H–X_ in the products. It is important to note that this crude assessment omits the impact of the role of BDFE_Au–Cl_ and BDFE_Au–OH_ in the starting complexes. Our crude assessment focuses only on the differences in product BDFE values, meaning we assume that BDFE_Au–Cl_ and BDFE_Au–OH_ are very close in energy. For free HCl, BDFE_H–Cl_ in the gas phase = 103 kcal/mol,^42^ while for the H_2_O ligand in [Au^II^(H_2_O)(terpy)]^2+^, we have estimated a value of 81 kcal/mol, yielding a 22 kcal/mol difference in product BDFE values. One caveat is that we do not have a BDFE_H–Cl_ value in DMF and that HCl and [Au^II^(H_2_O)(terpy)]^2+^ might not be the sole products obtained in this reaction. **1** and **2** displayed similar slope values in the plots that related BDFE_C–H_ and Δ*G*^⧧^ (0.39 and 0.30, respectively), which indicated a comparable HAT mechanism. A direct comparison can thus be made between the ratio of BDFE_H–X_ in the products (difference in thermodynamic driving force) and the ratio of the activation free energies for both oxidants:
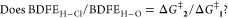


Such a comparison would allow us to assess if the increased value of BDFE_H–Cl_ results in a comparable and predictable reduction in activation energy for HAT compared to the metal–oxygen adduct. BDFE_H–Cl_/BDFE_H–O**(2)**_ = 1.27, while Δ*G*^⧧^_**2**_/Δ*G*^⧧^_**1**_ = 1.10 or 1.18 depending on the method used to calculate Δ*G*^⧧^ (Table 1). Given the inherent errors and generally large uncertainty in these measurements, the similarities in these ratios are striking.

We conclude that upon exchange of chloride for hydroxide, the stronger HCl bond imbues **1** with superior reaction rates, indicating that metal–chloride oxidants would generally yield kinetically more competent oxidants than analogous hydroxides.

An alternative explanation for the enhanced reactivity of **1** was that there was greater radical character on the Cl atom in **1** than on the O atom in **2**. Indeed, **1** showed a Δ*S*^⧧^ smaller than that of **2**, which might suggest that for **1** the reorganization of the vibrational levels after the HAT event was influenced less by the metal and more by the Cl ligand. Mader and co-workers observed large negative values of Δ*S*^⧧^ during HAT when Fe^II^ complexes bearing different ligands were involved but very small values when organic radicals were explored for the same type of oxidative mechanism.^35^ Zhang and co-workers explored the HAT reactivity of three Cu^III^–X complexes (X = F, Cl, or Br),^5^ where the Cu^III^–F complex showed a 200-fold higher rate of DHA oxidation compared to the other two. This was attributed to the higher radical character on F than Cl/Br, despite still reporting a very large and negative Δ*S*^⧧^ (−43 cal mol^–1^ K^–1^).

Starting from the respective single-crystal X-ray structures of **1** (Figure S2) and **2**,^43^ the geometry was optimized via DFT calculations using unrestricted BP86/def2-TZVPP (C, H, N, O, Cl)/SARC-ZORA-TZVPP (Au) with zeroth-order regular approximation and the COSMO(DMF) level of theory. The obtained geometries were in good agreement with the X-ray structures (Figures S34 and S35 and Tables S7 and S8). *Ab initio* CASSCF(8,5) calculations were conducted to shed light on the ligand field differences between **1** and **2** (see Figures S37 and S38). From this analysis, the Racah parameter *B* could be obtained to quantify the interelectronic repulsion.^44^ A decrease in the magnitude of *B* with respect to the free Au^III^ ion (*B*_0_) showed a correlation, known as the nephelauxetic effect. The ratio β = *B*/*B*_0_ gives an indication of charge transfer from the metal center to the ligand: the smaller the β, the greater the charge transfer and therefore more covalency is present in the metal–ligand bond.^45^ The parameter β is lower for **1** (0.49) than for **2** (0.64), which is in alignment with a decrease in the magnitude of the Mulliken atomic charges going from **2** (Au, 1.43; O, −0.77) to **1** (Au, 1.28; Cl, −0.46). This suggests a larger contribution of Au^II^–Cl^•^ together with the dominant Au^III^–Cl bond description for **1**.^44^ The electron-localized function (ELF) showed a minimum along the bond path between the Au cation and the oxygen of the hydroxide ligand in **2** (ELF at bond critical point of 0.23), whereas a higher likelihood of localization was observed along the bond path between Au and Cl in **1** [ELF at bond critical point of 0.29 (Figure S36)]. This result also indicates that the Cl ligand has more radical character due to a shift in the electron density of the Au–Cl bond from a purely ionic interaction (Au^III^···Cl^–^) toward covalent contributions (Au^II^–Cl^•^). These calculations suggest that the Cl atom in **1** may display marginally greater radical character that the O atom in **2**, potentially lowering the barrier to HAT in reactions with phenols.

We currently favor the former argument that the stronger H–Cl bond provides a greater thermodynamic driving force for HAT, lowering the activation barrier, simply because there is more experimental support for this postulate.

Finally, **1** also reacted with the hydrocarbons 1,4-cyclohexadiene (CHD) and 9,10-dihydroanthracene (DHA). For CHD, benzene was identified as the only product [yield of 105 ± 10% for a two-electron oxidation by **1** (Table S3)]. The ^1^H NMR spectra from this reaction also showed a shift and broadening of the residual signal attributed to H_2_O (δ = 3.34 ppm to δ = 3.72 ppm), suggesting that the increase in the water content upon reaction with CHD (Figures S39 and S40).^16^ The reaction with DHA [500 equiv, DMF, 25 °C (Figure S41)] showed a change in the electronic absorption spectrum with the formation of bands typical of anthracene (λ = 341, 360, and 379 nm), a two-proton, two-electron oxidized product. The plot of *k*_obs_ versus DHA concentration showed a linear correlation with the *k*_2_ of 0.046 M^–1^ s^–1^, which is slightly higher than that measured for **2** (0.020 M^–1^ s^–1^).^16^ The reaction was repeated using D_4_-DHA- (*k*_2_ = 0.018 M^–1^ s^–1^), yielding a primary kinetic isotope effect of 2.5 (Figure S42). The yield of anthracene was determined by GC-FID to be 80 ± 5%, when a two-electron reaction was considered (Table S3). We therefore concluded that **1** was a capable hydrocarbon oxidant at room temperature and reacted more rapidly than **2**. These observations for C–H activation are consistent with our kinetic and mechanistic analysis using phenols.

## Conclusions

In conclusion, we compared the oxidative reactivity of two high-valent oxidants differing only in their proton-accepting ligand, Cl^–^ (**1**) versus OH^–^ (**2**). The complexes were reacted under the exact same conditions. Both oxidants followed a HAT mechanism of C/O–H bond activation. The Au^III^–Cl complex reacted at superior rates with all substrates compared to the Au^III^–OH complex. We ascribed this to a greater thermodynamic driving force provided by the HCl product of oxidation by Au^III^–Cl. Our discoveries highlight the crucial role played by the proton-accepting ligands in tuning the reactivity of high-valent oxidants and demonstrate that metal–halide oxidants can yield lower activation barriers to oxidative C/O–H activation compared to those of their corresponding metal–oxygen adducts.

## Supporting Information Available

Reactivity protocols and results, EPR analysis, NMR analysis, and DFT and quantum chemical calculations (PDF)

## Accession Codes

CCDC 2079645 contains the supplementary crystallographic data for this paper. These data can be obtained free of charge via www.ccdc.cam.ac.uk/data_request/cif, or by emailing data_request@ccdc.cam.ac.uk, or by contacting The Cambridge Crystallographic Data Centre, 12 Union Road, Cambridge CB2 1EZ, UK; fax: +44 1223 336033.
