# Supplementary material for: Comparing Metal–Halide and −Oxygen Adducts in Oxidative C/O–H Activation: AuIII–Cl versus AuIII–OH
Source: Inorg Chem. 2021 Sep 28;60(20):15610–6. doi: 10.1021/acs.inorgchem.1c02222 (PMC8527453; doi:10.1021/acs.inorgchem.1c02222)
Supplement: Supplementary file 1 — ic1c02222_si_001.pdf [file ic1c02222_si_001.pdf]

Supporting Information

for

Comparing metal-halide and -oxygen adducts in oxidative C/O–H activation: Au^{III}–Cl versus Au^{III}–OH

Marta Lovisari,[a] Robert Gericke,[b] Brendan Twamley,[a] and Aidan R. McDonald*[a]

[a] Marta Lovisari, Dr. Aidan R. McDonald, School of Chemistry, Trinity College Dublin, The University of Dublin, College Green, Dublin 2, Ireland. Email: aidan.mcdonald@tcd.ie

[b]Dr. Robert Gericke, Current address: Helmholtz-Zentrum Dresden-Rossendorf e. V., Institute of Resource Ecology Bautzner Landstraße 400, 01328 Dresden, Germany

Experimental Procedures

Materials

All solvents and reagents were purchased from commercial sources and used as received, unless otherwise stated. Complex **1** was synthesised as reported.[1] Either $KAuCl_4$ or $HAuCl_4$ were used as starting materials for the synthesis of **1**, they were purchased from Sigma Aldrich or Fisher Scientific and used as received. p-X-2,6-di-*tert*-butylphenols were recrystallized from hexane or ethanol/water prior to use. For reactions performed in the absence of oxygen, solvents (N,N-dimethylformamide, tetrahydrofuran, diethyl ether) were freezed-pump-thawed three times and stored in a nitrogen atmosphere glovebox. TEMPOH (1-hydroxy-2,2,6,6-tetramethyl-piperidine), 4-CH_3O-TEMPOH (4-methoxy-1-hydroxy-2,2,6,6-tetramethyl-piperidine) and 4-oxo-TEMPOH (4-oxo-1-hydroxy-2,2,6,6-tetramethyl-piperidine) were synthesized according to reported procedures.[2] 4-CH_3O-*[D]*-2,6-DTBP was synthesized according to a reported procedure.[3] *[D]*$_4$-9,10-dihydroanthracene (*[D]*$_4$-DHA) was synthesized according to a reported procedure (deuteration degree = 97% by ^{1}H-NMR).[4]

Physical Methods

^{1}H and ^{13}C{^{1}H} nuclear magnetic resonance (NMR) analyses were performed on an Agilent MR400 instrument (400.13 MHz for ^{1}H NMR, 100.61 MHz for ^{13}C{^{1}H} NMR). The signals were internally referenced to tetramethylsilane. Electronic absorption spectra were recorded on a Hewlett Packard (Agilent) 8453 diode array spectrophotometer. Electrospray ionization (ESI) mass spectra were acquired using a Micromass time of flight spectrometer (TOF), interfaced to a Waters 2690 HPLC. Attenuated total reflectance infra-red (ATR-FTIR) spectra were recorded on a Perkin-Elmer Spectrum 100 Fourier transform infrared spectrometer.

Crystallographic analysis

Crystals suitable for single crystal X-ray diffraction measurements were grown over two days from a N,N-dimethylformamide (DMF)/ diethyl ether (Et_2O) mixture. Data were measured (λ = 0.71073 Å) at 100(2)K on a Bruker D8 Quest Eco with an Oxford Cryostream low temperature device using a MiTeGen micromount. Bruker APEX [5] software was used to collect the data and correct for Lorentz and polarization effects. Data were integrated with SAINT[6] and corrected for absorption effects using the Multi-Scan method, SADABS.[7] The structure was solved with the XT[8] structure solution program using Intrinsic Phasing and refined with the XL[9] refinement package using Least Squares minimization with Olex2,[10] using the space group $P2_1$, with Z = 2. This confirms the desired structure with formula unit, $C_{15}H_{11}AuCl_3N_3O_8$, with slightly different cell parameters with respect to those already reported [11].[6] The structure was refined as an inversion twin, with a refined Flack parameter of 0.011(6). One perchlorate displays disorder in one oxygen site which was modelled in two orientations with the majority population 0.58(4) using geometric (SADI) and displacement (ISOR, SIMU) restraints. The growth of such crystals over two days showed that was **1** stable in dry DMF, allowing its reactivity to be explored in such conditions. Crystallographic data for this paper have been deposited with the Cambridge Crystallographic Data Centre as supplementary publication no. 2079645. Copies of the data can be obtained, free of charge, on application to CCDC, 12 Union Road, Cambridge CB2 1EZ, UK, (fax: +44-(0)1223-336033 or e-mail:deposit@ccdc.cam.ac.uk).

EPR analysis

Electron paramagnetic resonance (EPR) spectra of frozen solutions were acquired on a Bruker EMX X-band EPR, equipped with an Oxford Instruments CE 5396, ESR9 Continuous Flow Cryostat, a precision Temperature Controller and an Oxford Instruments TTL20.0/13 Transfer Tube. EPR samples were prepared by freezing the EPR tubes containing the analyte solutions, previously prepared at the UV-Vis spectrophotomer, in liquid nitrogen. EPR spectra of the 2,4,6-tris-*tert*-butylphenoxyl radical were recorded at 77 K, 9.2 GHz, 0.2 mW microwave power, with a 10 mT field sweep in 84 s, and 0.2 mT field modulation amplitude. EPR spectra of 4-X-TEMPO were recorded at 77 K, 9.2 GHz, 2.02 mW microwave power, with a 60 mT field sweep in 84 s, and 0.3 mT field modulation amplitude. Integration, simulation, and fitting were performed with Matlab and the easySpin computational package. [12] The simulation for the phenoxyl radicals spectra was modelled as an S = ½ electron spin with an isotropic g tensor. The oxidation yield of the samples was calculated by quantification of the concentration of spin in the samples. This was obtained by comparison of the double integral

of the signals to that of a frozen reference 3 mM solution of (2,2,6,6-tetramethyl-piperidin-1-yl)oxyl (TEMPO), measured under the same conditions.

Electrochemistry

Cyclic voltammetry (CV) experiments were conducted with a CH Instruments 600E electrochemical analyzer, using a glassy carbon working electrode, a platinum wire counter electrode and an Ag/$AgNO_3$ 0.01 M reference electrode. Cyclic voltammetry experiments were conducted on a 1.0 mM solution of **1** in DMF and on a 1.0 mM solution of **2H⁺** in DMF at room temperature, with a 0.05, 0.1 and 0.2 V s^{-1} under an argon atmosphere. In both experiments 0.1 M $^{n}Bu_4NPF_6$ was used as the supporting electrolyte. Potentials were referenced against the Fc^+/Fc couple (Fc = ferrocene). The voltammograms of Fc were acquired under the same conditions.

Gas chromatography-Flame Ionisation Detector (GC-FID) analysis

Gas chromatography experiments have been performed using a ThermoFisher TRACE™ 1300 Gas Chromatograph equipped with a Flame Ionisation Detector. Hydrogen was provided by a Parker Hydrogen Gas Generator 20H-MD. Air was provided by Parker Zero Air Generator UHP-10ZA-S. The column used was a ThermoFisher TraceGOLD TG-1MS GC column. Post-reaction solutions from the reaction of **1** with different substrates were prepared at 25 °C. Different amounts of substrate (4-CH_3O-2,6-DTBP, 2,4,6-TTBP, 2,6-DTBP, DHA) were added as DMF solutions under continuous stirring until completion of the reaction, after which the mixture was analysed by GC-FID. A reference standard solution was prepared by adding the same amount of equivalents of substrate to 2 mL of pure DMF. The instrument method for anthracene was based on a temperature ramp (2 min at 175 °C, 10 °C/min to 250 °C, 2 min at 250 °C) with splitless injections of 1 µL each. Under these conditions, the retention time for anthracene was between 6.3-6.4 min. The instrument method for 2,6-di-*tert*-butylquinone (2,6-DTBQ) was based on a temperature ramp (2 min at 75 °C, 10°C/min to 200 °C, 2 min at 200 °C) with splitless injections of 1 µL each. Under these conditions, the retention time for 2,6-di-*tert*-butylquinone was between 12.3-12.4 min. The quantitative analysis of the analytes was based on an external calibration curve (reported below). Yields were calculated as an average of four different solutions containing the same number of equivalents of substrate and repeating it for 2-3 different substrates concentrations.

Quantum chemical calculations

The geometry optimizations of **1** and **2** were carried out with ORCA 4.1.2 using DFT unrestricted BP86 functional, def2-TZVPP basis set for C, H, N, O, Cl and SARC-ZORA-TZVPP for Au atoms and atom pairwise dispersion correction with Becke-Johnson damping scheme (D3BJ). Calculations were started from the molecular structures of **1** and **2** obtained by single crystal X-ray diffraction. From the DFT obtained geometries, the CASSCF(8,5) orbitals were pre-converged using a much smaller basis set SV for C, H, N, O, Cl and SARC-ZORA-TZVPP for Au only in singlet sate (nroots = 15) without COSMO(DMF). *Ab initio* ligand field analysis were performed at stage-average CASSCF(8,5) level of theory using the def2-TZVPP basis set for C, H, N, O, Cl and SARC-ZORA-TZVPP for Au in singlet (nroots 15) and triplet (nroots 10) states including COSMO(DMF). Mulliken atomic charges and ELF calculations were performed on the final CASSCF(8,5) wavefunction using MultiWFN.[13] Graphics were generated using ChemCraft.[14]

Equilibrium constant calculations

We adopted a method reported by Mayer and co-workers to determine K_a.[15] Sub-stoichiometric equivalents of PyHOTf were added to **2** and the absorptivity changes were monitored. For the plot of [$\mathbf{2H^+}$][Py]/[**2**] against [PyHOTf], [$\mathbf{2H^+}$][Py]/[**2**] was calculated as follows: (A – A_0**2**)/($A_0$$\mathbf{2H^+}$ - A) where A = absorbance intensity at λ = 369 nm, A_0**2** = absorbance intensity at λ = 840 nm at 100% of **2**; $A_0$$\mathbf{2H^+}$ = absorbance intensity at λ = 840 nm at 100% of $\mathbf{2H^+}$; [Py] = (A – A$\mathbf{2}_0$**H+**)/(A_0**2H+** - A_0**2**); [PyHOTf] = total [PyHOTf] added - [Py].

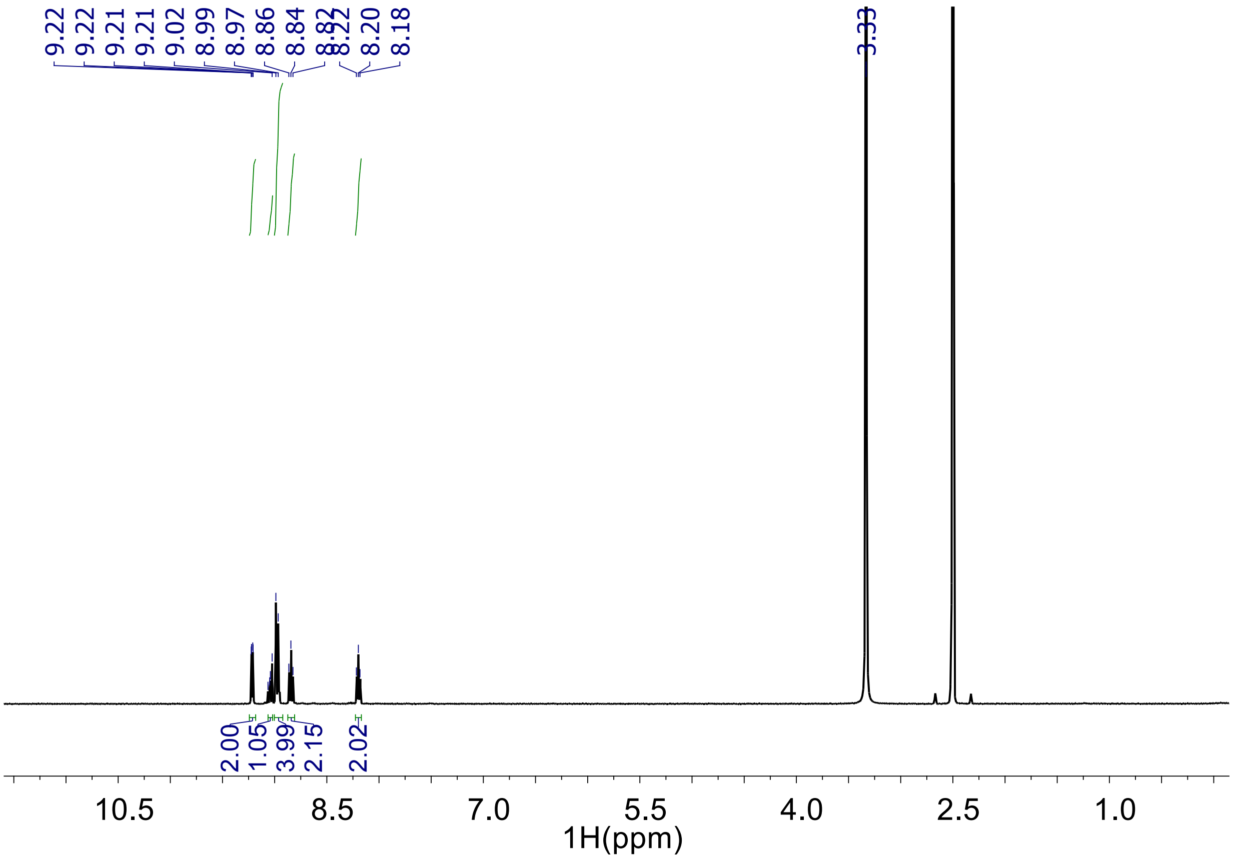

Figure S1. ^{1}H-NMR spectrum (400 MHz, DMSO-D_6) of **1**.

Table S1. Crystal data and structure refinement for 1.

$*R_1 = \Sigma||Fo| - |Fc||/\Sigma|Fo|$, $wR_2 = [\Sigma w(F_o^2 - F_c^2)^2 / \Sigma w(F_o^2)^2]^{1/2}$

CCDC no.	2079645	ρ_{calc} (g/cm^3)	2.388
Empirical formula	$C_{15}H_{11}AuCl_3N_3O_8$	μ (mm^{-1})	8.446
Formula weight	664.58	F(000)	632
Temperature (K)	100(2)	Crystal size (mm^3)	0.25 x 0.15 x 0.08
Crystal system	Monoclinic	Radiation	MoKα (λ = 0.71073 Å)
Space group	$P2_1$	2Θ range for data collection (°)	2.931 to 28.359
a (Å)	8.1167(4)	Index ranges	-10≤h≤10, -18≤k≤18, -11≤l≤11
b (Å)	13.8166(6)	Reflections collected	17712
c (Å)	8.3177(4)	Independent reflections	4568 [R(int) = 0.0231]
α (°)	90	Data/restraints/parameters	4568 / 98 / 283
β (°)	97.7580(10)	Goodness-of-fit on F^2	0.917
γ (°)	90	Final R* indices [I>2σ(I)]	R1 = 0.0141, wR2 = 0.0325
Volume (Å^3)	924.25(8)	R indices (all data)	R1 = 0.0156, wR2 = 0.0329
Z	2	Largest diff. peak and hole (e.Å^{-3})	0.944 /-0.731

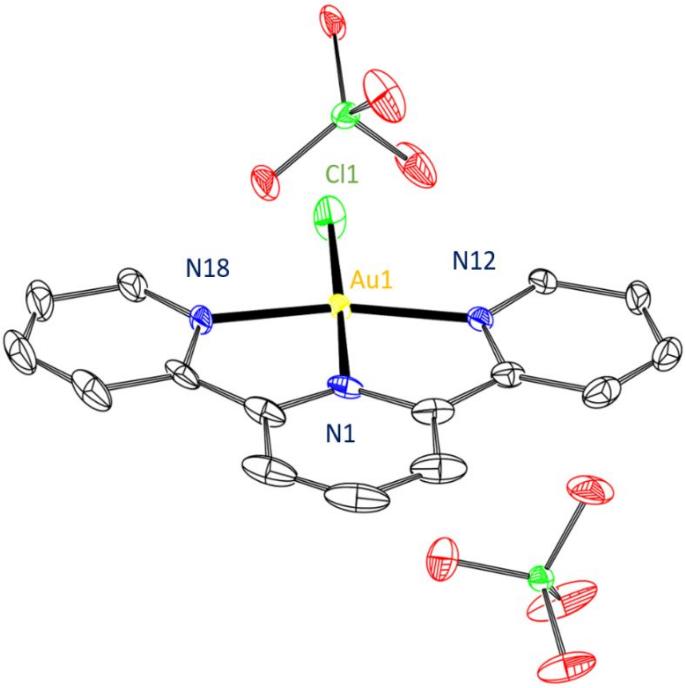

Figure S2. Crystal structure of 1. Thermal ellipsoids are represented at 50% probability. Hydrogen atoms and disorder in the perchlorate counterions are not depicted for clarity.

Table S2. Selected bond distances for Au^{III}–Cl complexes supported by terpyridine ligands.

Complex	Au(1)–Cl(1) (Å)	N_{ax}-Au(1) (Å)	N_{eq}–Au(1)-Cl(1) (°)	N_{eq}–Au(1)-N_{ax} (°)	Reference
**1**	2.2544(13)	1.944(5)	99.23(14)<br>97.54(18)	81.8(2)<br>81.37(15)	This work
$[Au^{III}(Cl)(terpy)]Cl_2$	2.269(2)	1.931(7)	98.7(2)<br>98.5(2)	81.4(3)<br>81.4(3)	[16]
$[Au^{III}(Cl)(4\text{-MeOPh-terpy})](PF_6)_2$	2.2559(24)	1.924(6)	-	80.1(3)<br>82.5(3)	[17]
$[Au^{III}(Cl)(4\text{-2Py-terpy})](PF_6)_2$	2.257(3)	1.938(8)	98.5(2)<br>98.6(3)	81.6(3)<br>81.3(3)	[18]
$[Au^{III}(Cl)(4\text{-3Py-terpy})](PF_6)_2$	2.257(3)	1.971(7)	99.6(2)<br>98.5(3)	79.8(3)<br>82.1(3)	[18]

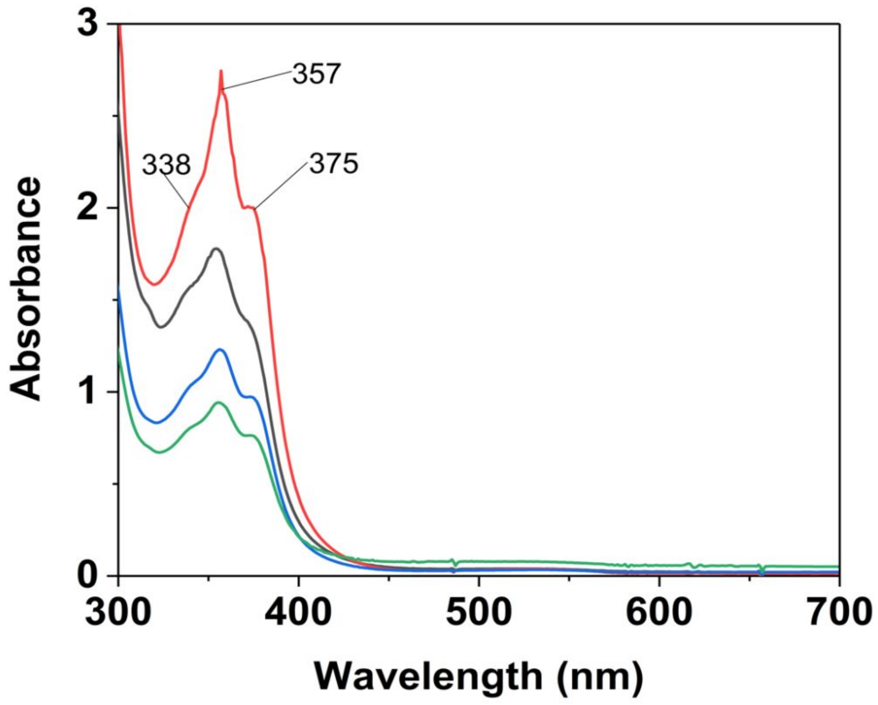

Figure S3. Electronic absorption spectra (0.035, 0.07, 0.125 and 0.25 mM, DMF, 25 ℃) of **1**.

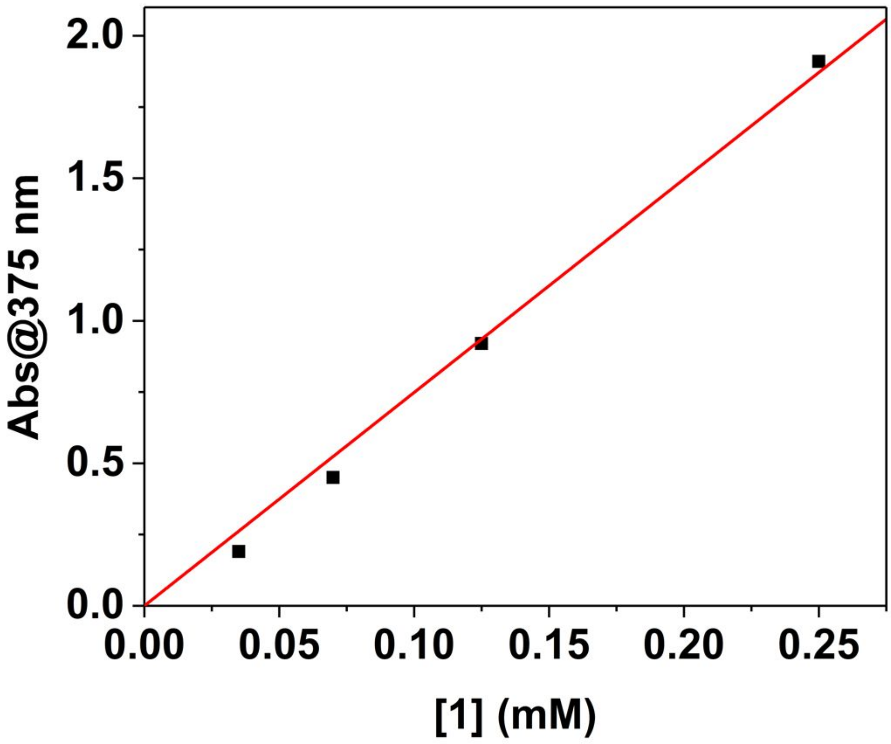

Figure S4. Plot of the absorbance vs. concentration for **1** at λ = 375 nm.

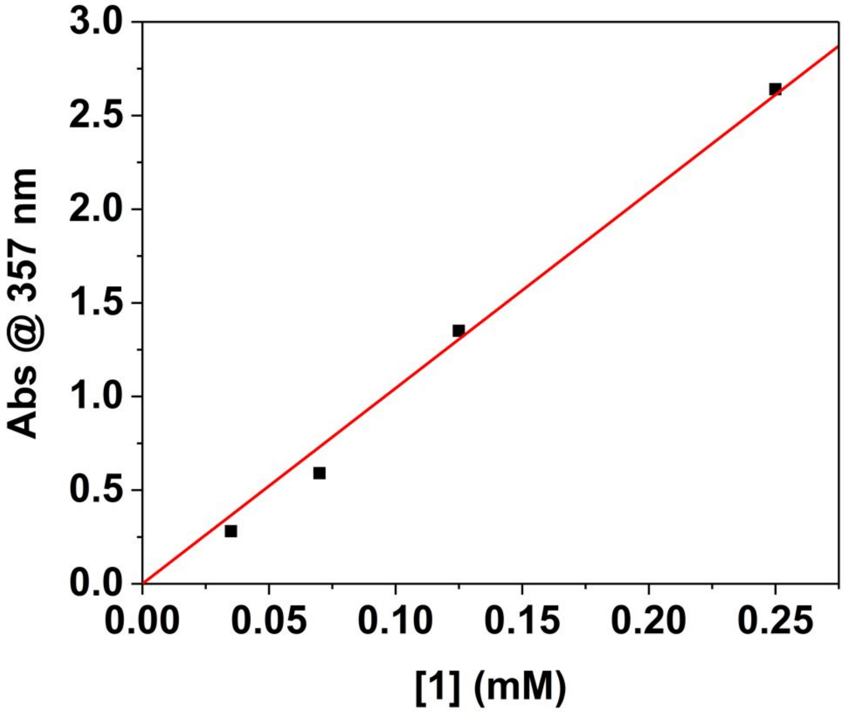

Figure S5. Plot of the absorbance vs. concentration for **1** at λ = 357 nm.

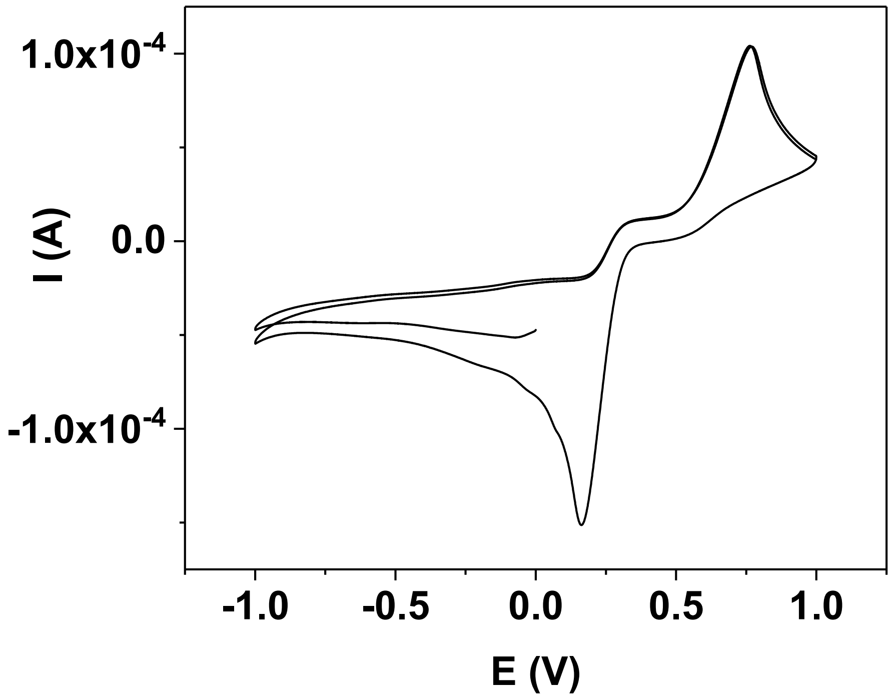

Figure S6. Cyclic voltammetry of **1** in DMF, supporting electrolyte: $^{n}Bu_4NPF_6$ 0.1 M, working electrode: carbon, counter electrode: Pt wire, reference electrode: Ag/$AgNO_3$, scan rate: 0.1 V s^{-1}.

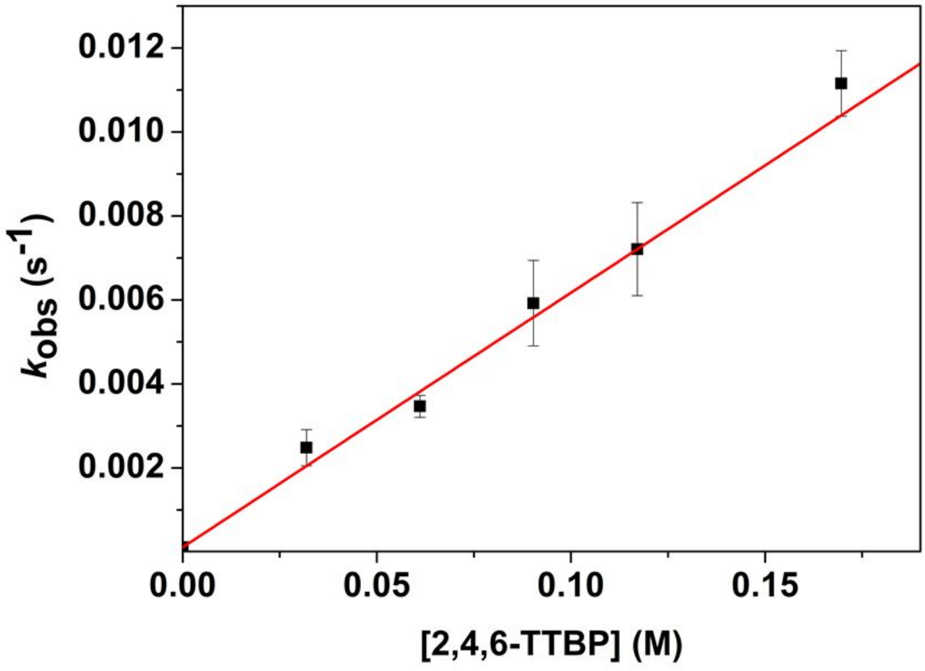

Figure S7. Plot of k_{obs} against [2,4,6-TTBP] determined for the reaction between **1** and 2,4,6-TTBP.

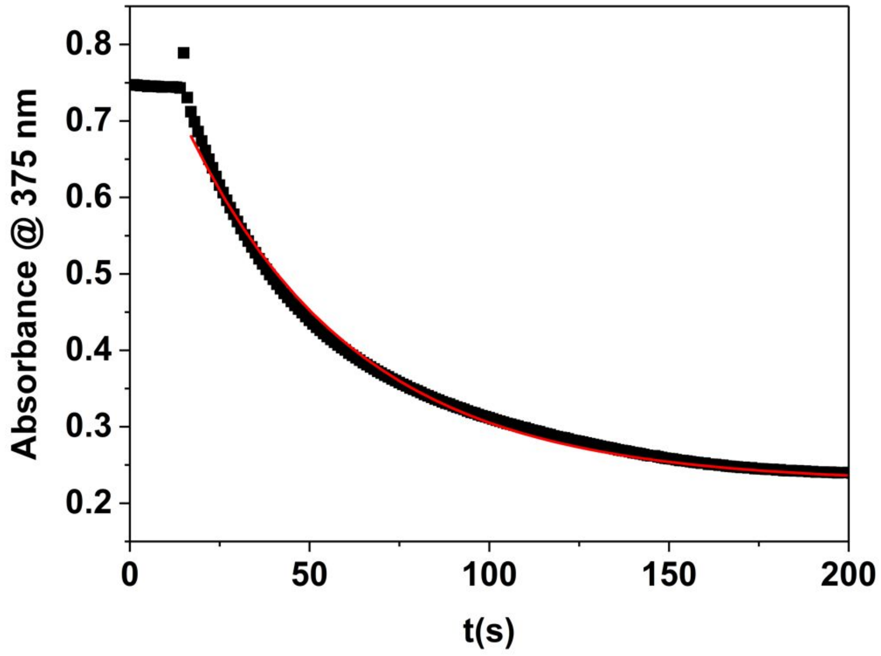

Figure S8. Plot of the absorbance at λ = 375 nm against time during the reaction between **1** and 4-CH3O-2,6-DTBP (100 equiv.) at 25 °C in DMF.

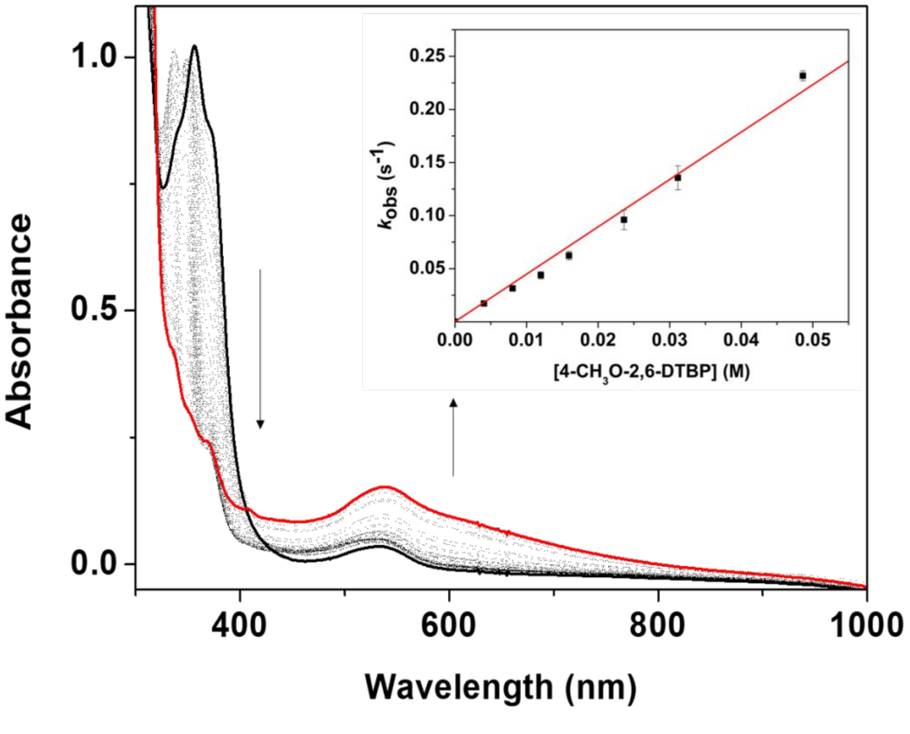

Figure S9. Electronic absorption spectra of the reaction of **1** (0.1 mM, 25 °C, DMF) with 4-CH_3O-2,6-DTBP (100 equiv.). Black trace = 0 s, red trace = 1000 s. Inset: plot of k_{obs} against [4-CH_3O-2,6-DTBP] determined for the reaction between **1** and 4-CH_3O-2,6-DTBP.

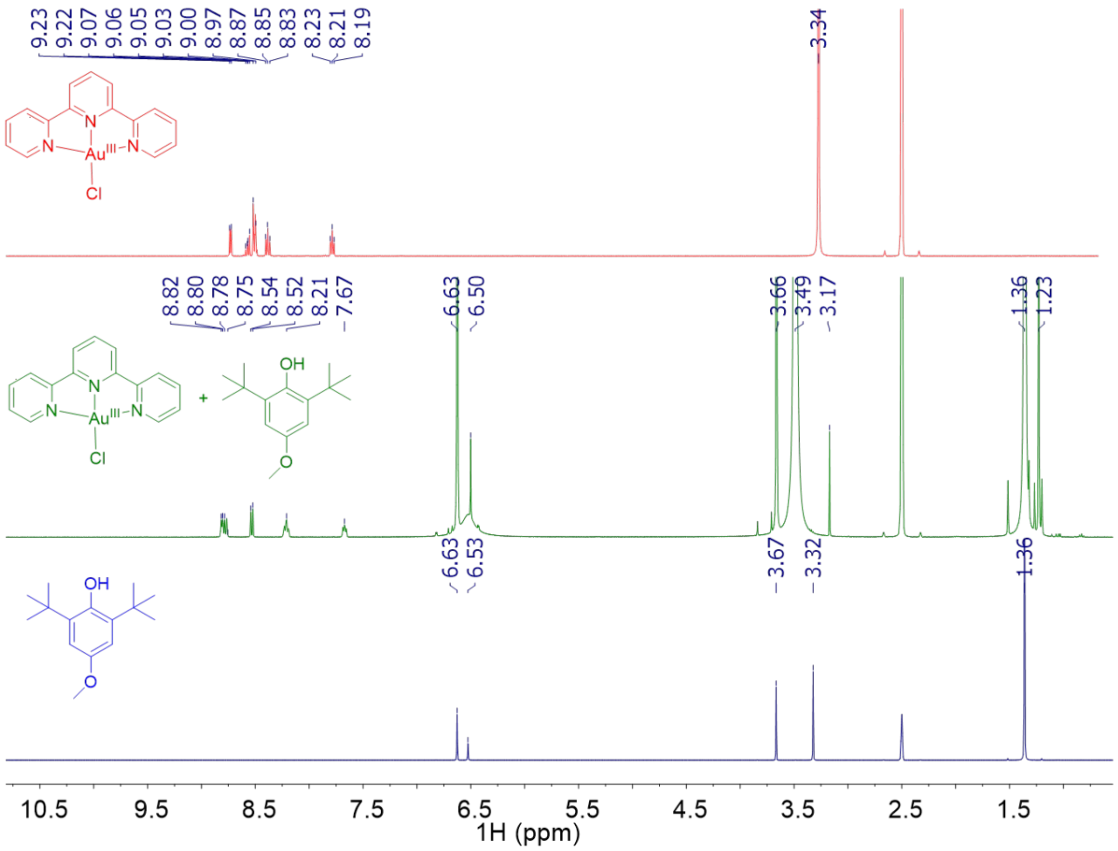

Figure S10. ^{1}H NMR (400 MHz, DMSO-D$_6$) of the reaction between **1** and 25 equiv. 4-CH_3O-2,6-DTBP. Red = complex 1, Blue = 4-CH_3O-2,6-DTBP, Green = complex 1 in presence of 25 equiv. 4-CH_3O-2,6-DTBP after ~4' from the mixing of the reagents**.**

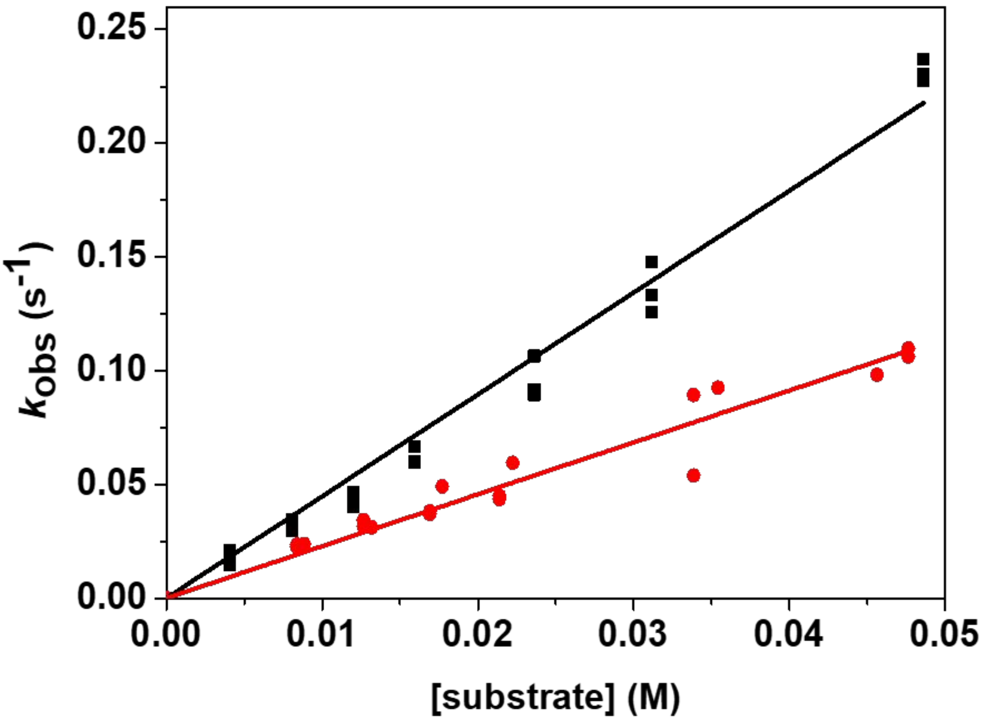

Figure S11. Plot of k_{obs} versus [substrate] determined for the reaction between complex **1** and 4-proto-CH_3O-2,6-DTBP (black trace) and 4-deutero-CH_3O-2,6-DTBP (red trace).

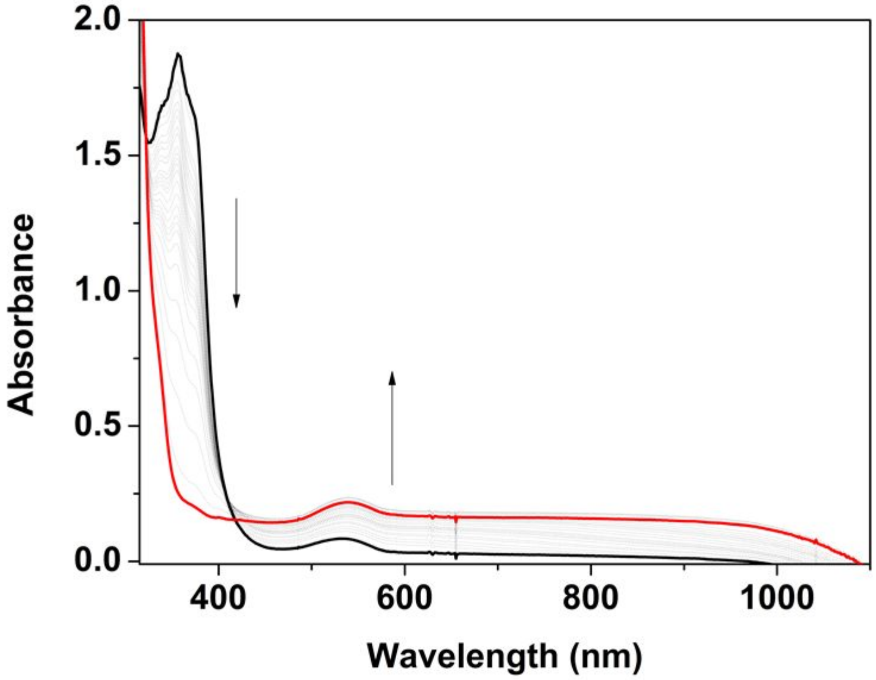

Figure S12. Electronic absorption spectra of the reaction of **1** (DMF, 25 °C, 0.17 mM) with 4-CH_3-2,6-DTBP (500 equiv.) monitored for 3000 s. black trace = 0 s, red trace = 3000 s.

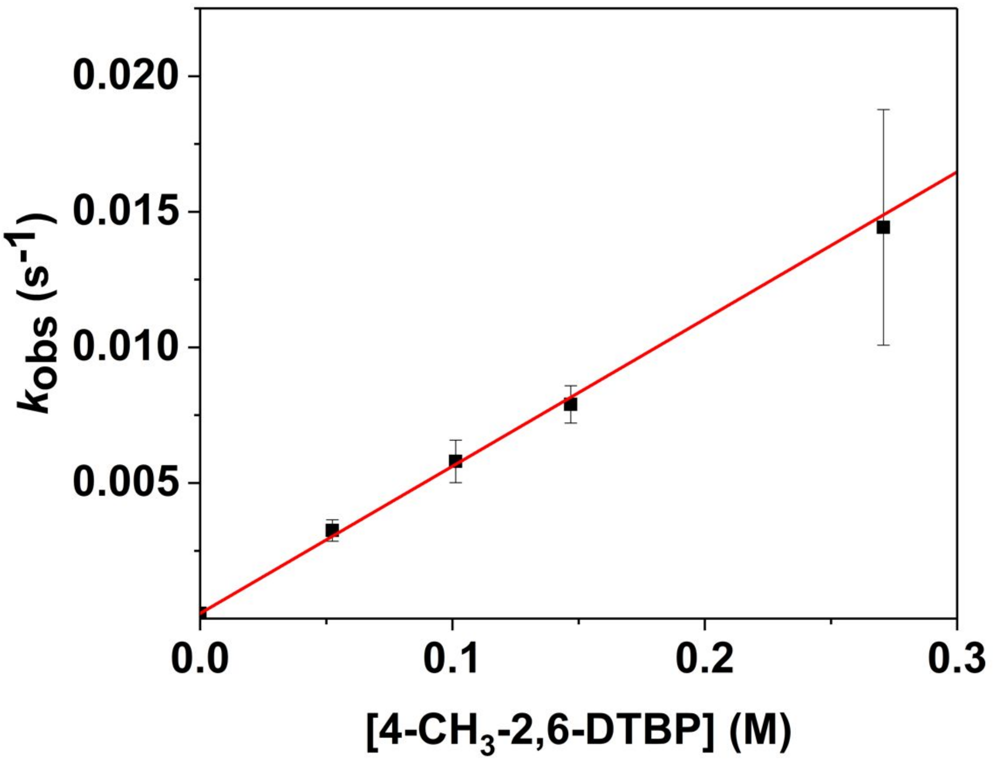

Figure S13. Plot of k_{obs} against [4-CH_3-2,6-DTBP] determined for the reaction between **1** and 4-CH_3-2,6-DTBP.

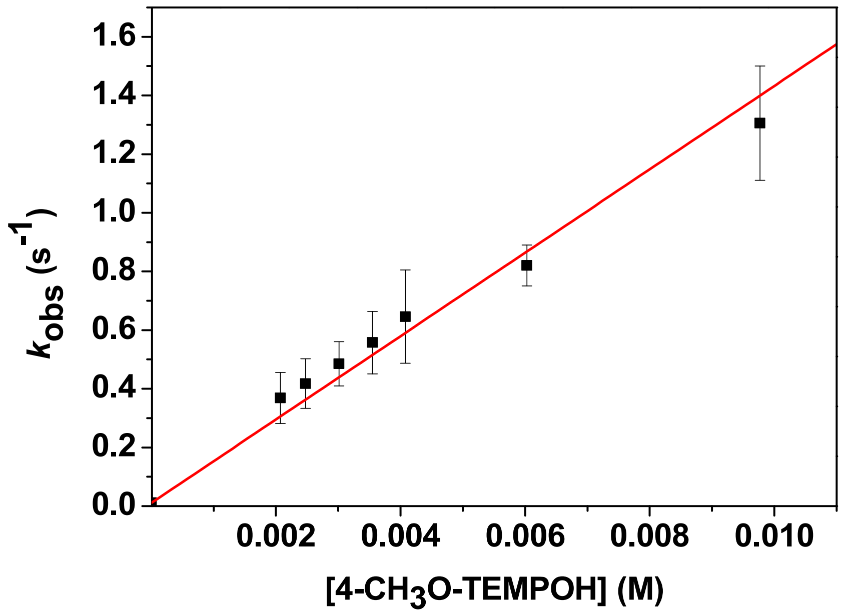

Figure S14. Plot of k_{obs} against [4-CH_3O-TEMPOH] determined for the reaction between **1** and 4-CH_3O-TEMPOH.

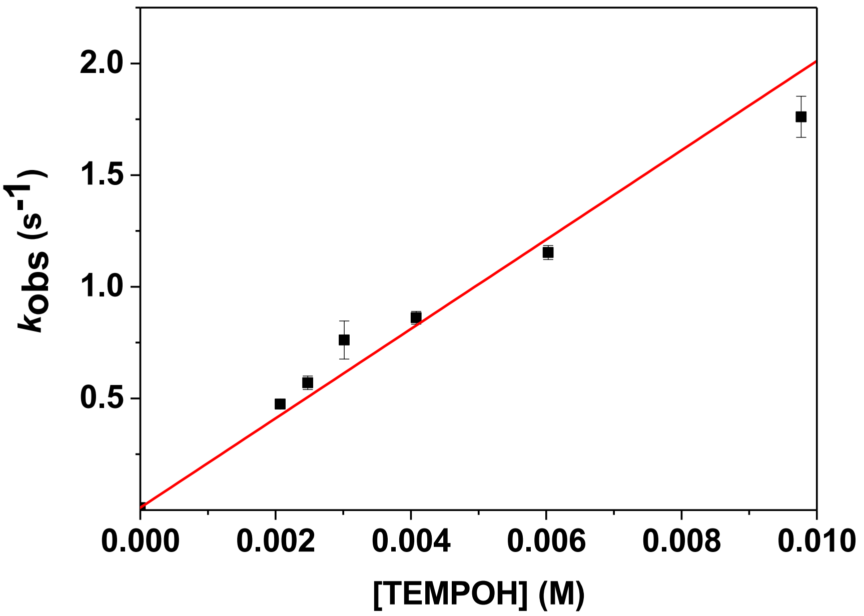

Figure S15. Plot of k_{obs} against [TEMPOH] determined for the reaction between **1** and TEMPOH.

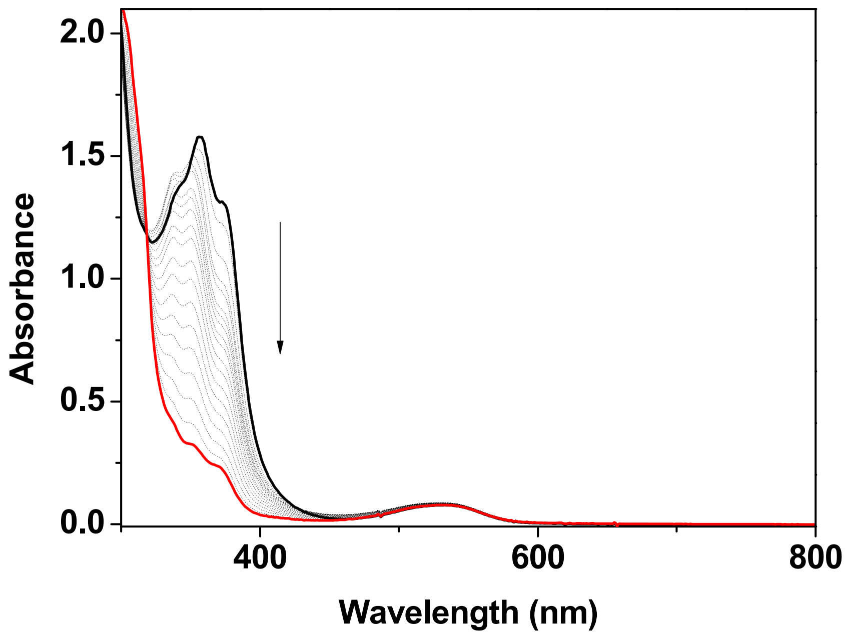

Figure S16. Electronic absorption spectra of the reaction of **1** (DMF, 25 °C, 0.15 mM) with 4-oxo-TEMPOH (20 equiv.) monitored for 100 s. black trace = 0 s, red trace = 100 s.

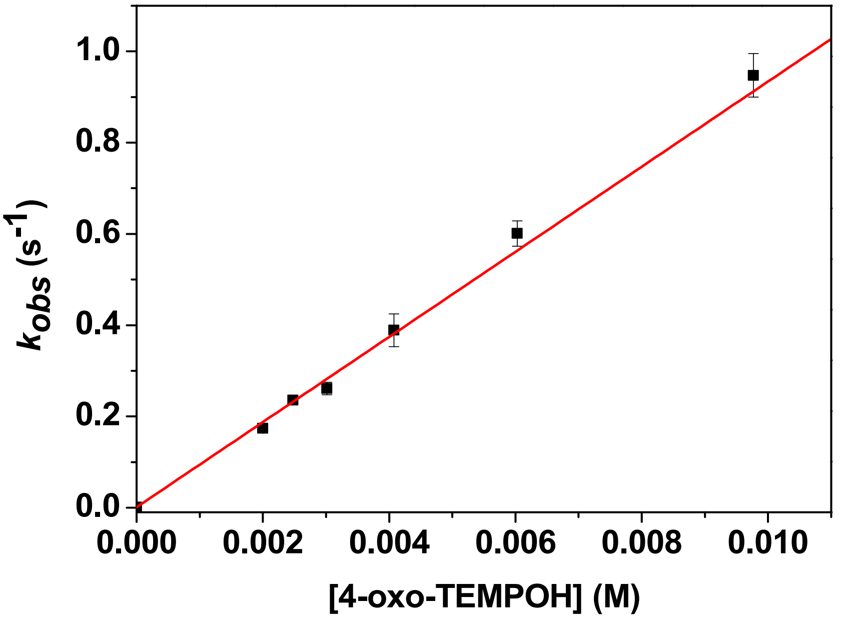

Figure S17. Plot of k_{obs} against [4-oxo-TEMPOH] determined for the reaction between **1** and 4-oxo-TEMPOH.

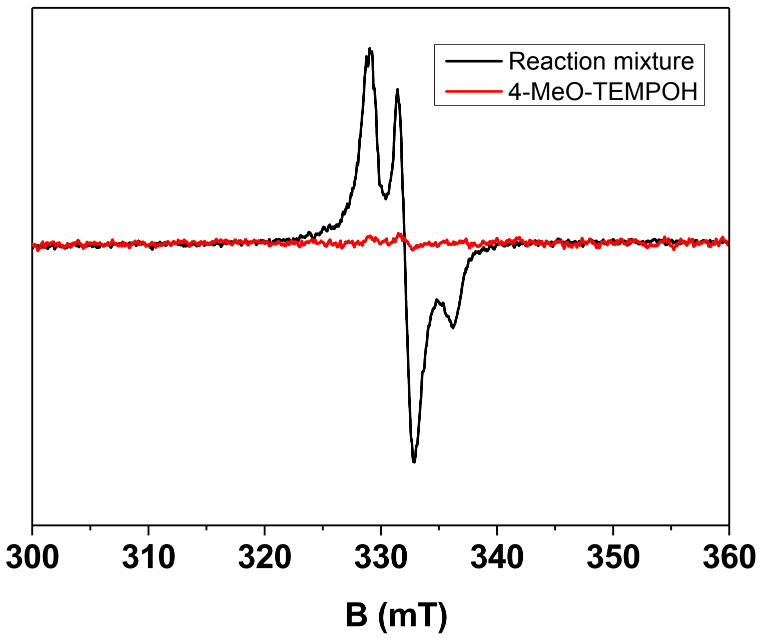

Figure S18. X-band EPR spectra of the reaction mixture of **1** with 4-CH_3O-TEMPOH (black trace) and of 4-CH_3O-TEMPOH alone (red trace) acquired from frozen DMF solutions at 77 K with a 2.01 mW microwave power and a 0.3 mT modulation amplitude.

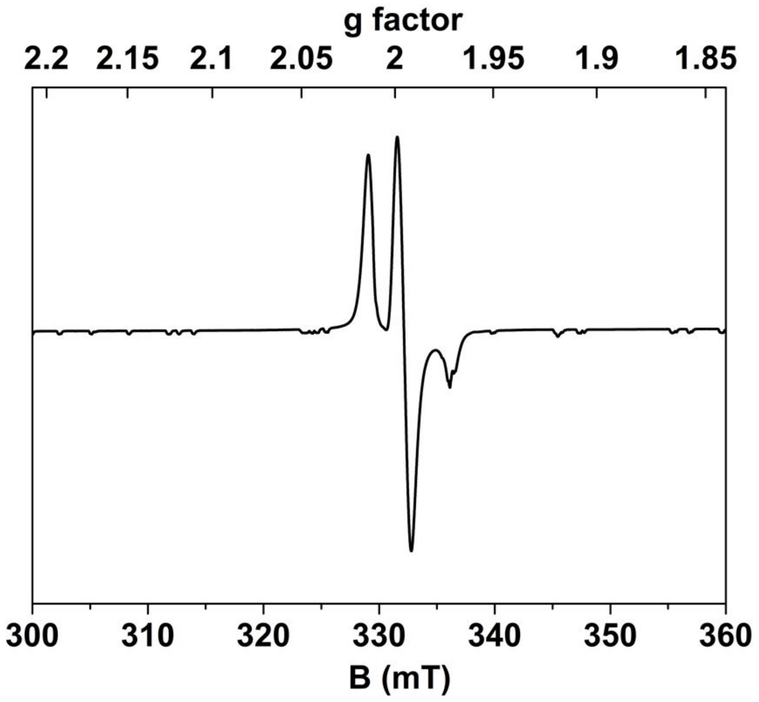

Figure S19. X-Band EPR spectrum of the reaction mixture of **1** with 4-oxo-TEMPOH (50 equiv.) after 100 s. The spectrum was acquired from a frozen DMF solution and measured at 77 K with a 1.98 mW microwave power and a 0.3 mT modulation amplitude.

Table S3. Yields of oxidation products for complex **1** with different substrates.

Substrate	Product	Stoichiometry	Yield (%)	Method
4-CH_3O-2,6-DTBP	phenoxyl radical	1-electron	-	EPR
			-	UV-Vis
	2,6-DTBQ	2-electrons	96±7	GC-FID
2,4,6-TTBP	phenoxyl radical	1-electron	75±20	EPR
			80±12	UV-Vis
	2,6-DTBQ	2-electrons	71±6	GC-FID
2,6-DTBP	TTBTD	2-electrons	10 ± 1	UV-Vis
TEMPOH	TEMPO	1-electron	77±20	EPR
4-CH_3O-TEMPOH	4-CH_3O-TEMPO	1-electron	82±20	EPR
4-oxo-TEMPOH	4-oxo-TEMPO	1-electron	84±20	EPR
CHD	benzene	2-electrons	105±10	^{1}H-NMR
DHA	anthracene	2-electrons	80±5	GC-FID

Table S4. Kinetic parameters for the reaction between **1** and substrates bearing O–H bonds.[19, 20]

Substrate	k_2 (M^{-1} s^{-1})	$BDFE_{O–H}$ (kcal/mol)[a]
4-oxo-TEMPOH	93.28	65.6
TEMPOH	200.02	66.5
4-CH_3O-TEMPOH	142.15	65.0
[*H*]-4-CH_3O-2,6-DTBP	4.48	*74*
[*D*]-4-CH_3O-2,6-DTBP	2.34	-
4-CH_3-2,6-DTBP	0.053	*77*
2,4,6-TTBP	0.056	77.1

[a] The values in italics are estimated based on those reported in other solvents.

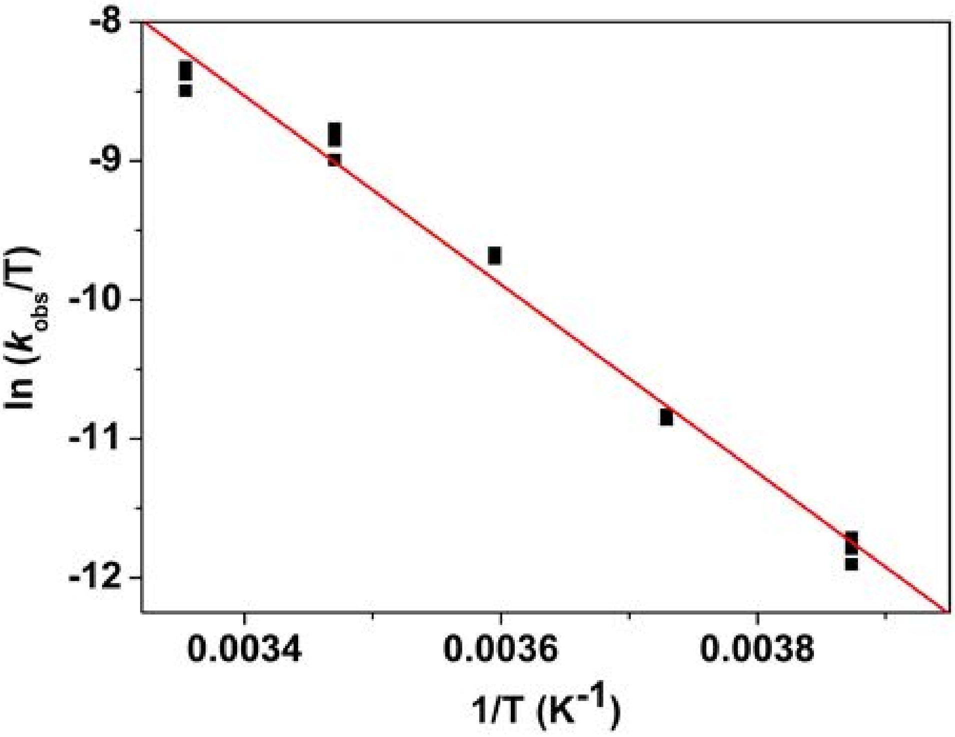

Figure S20. Plot of ln(k_{obs}/T) versus 1/T for **1** in its reaction with 4-CH_3O-2,6-DTBP. $\Delta H^{‡}$= 12.5 kcal/mol; $\Delta S^{‡}$= -18 cal $mol^{-1}K^{-1}$.

Table S5. Acids screened for reaction with **1** in DMF at 25 °C. The reported pK_a values are in H_2O.

Acid	pK_a in H_2O	Reaction?
HCl	-5.9	Yes, apparent decomplexation
p-toluensulfonic acid	-2.8	Yes
Trifluoroacetic acid	0.52	Yes
Nitrobenzoic acid	3.41	Yes
Formic acid	3.75	Yes
Benzoic acid	4.19	Yes
Acetic acid	4.75	Yes
Pyridinium triflate	5.21	No
Dimethylformamidium triflate	6.1	No

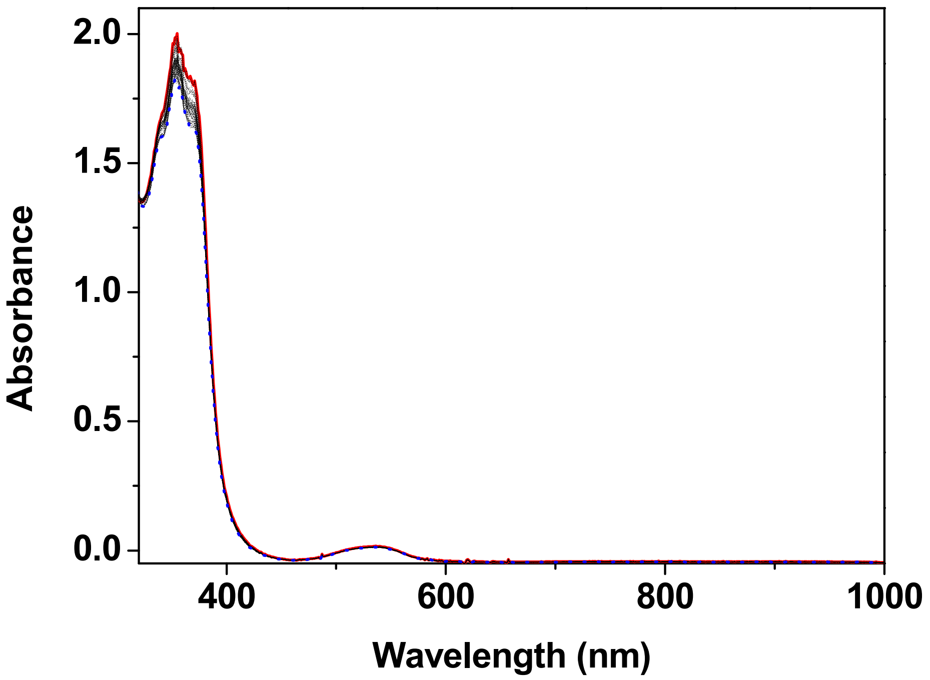

Figure S21. Change in the electronic absorption spectra of **1** upon addition of 20 equiv. benzoic acid (red trace, 20 equiv. benzoic acid).

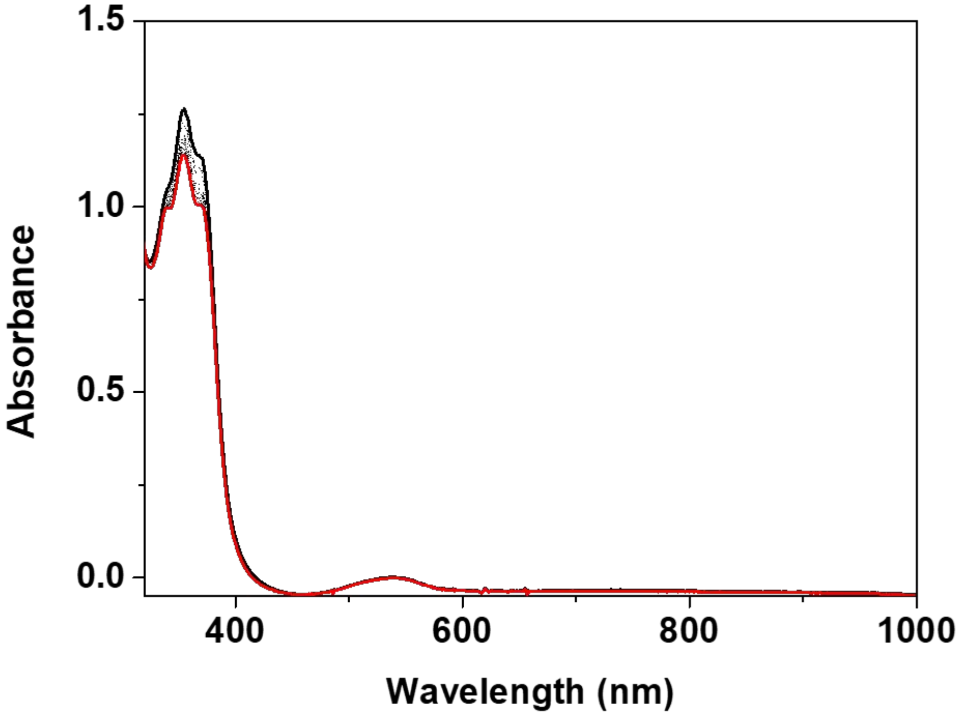

Figure S22. Change in the electronic absorption spectra of **1** upon addition of 20 equiv. acetic acid (red trace, 20 equiv. acetic acid).

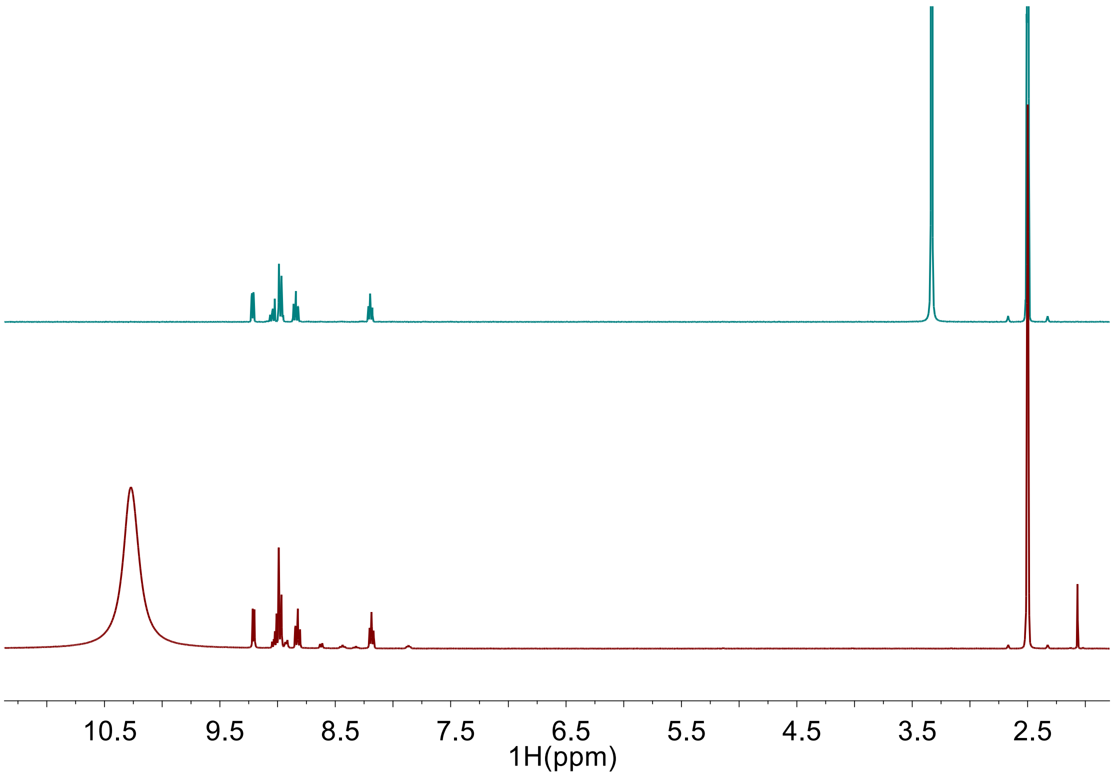

Figure S23. ^{1}H-NMR spectra (400 MHz, DMSO-D$_6$) of **1** before (green trace) and after (red trace) the addition of 20 equiv. of trifluoroacetic acid. The peak shifted from 3.4 to 10.2 ppm is assigned to protonated H_2O.

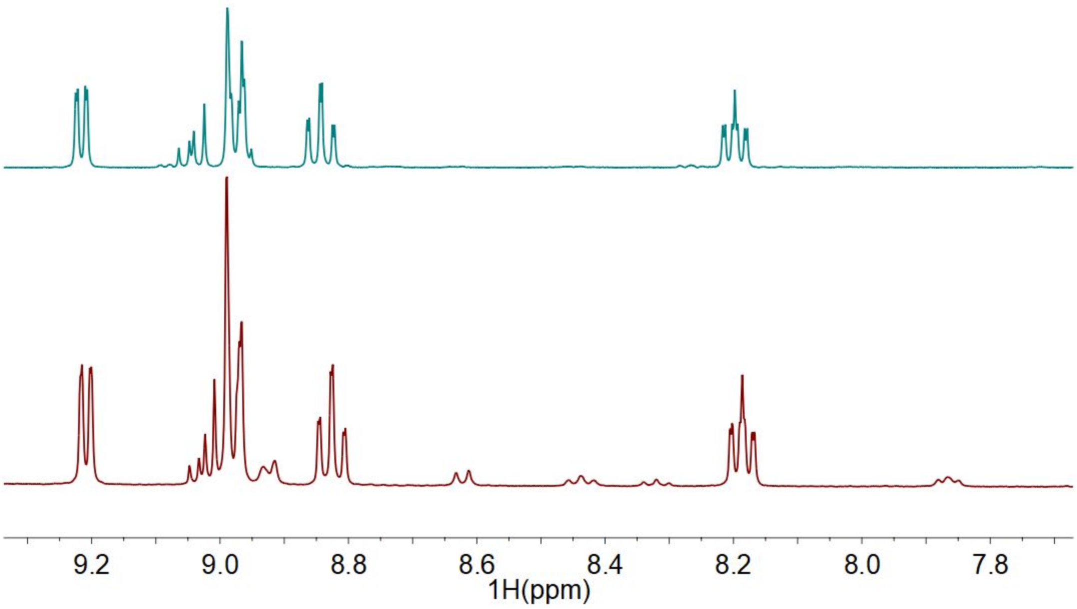

Figure S24. Selected region between 7.7 and 9.5 ppm of the ^{1}H-NMR spectra (400 MHz, DMSO-D$_6$) of **1** before (green trace) and after (red trace) the addition of 20 equiv. of trifluoroacetic acid.

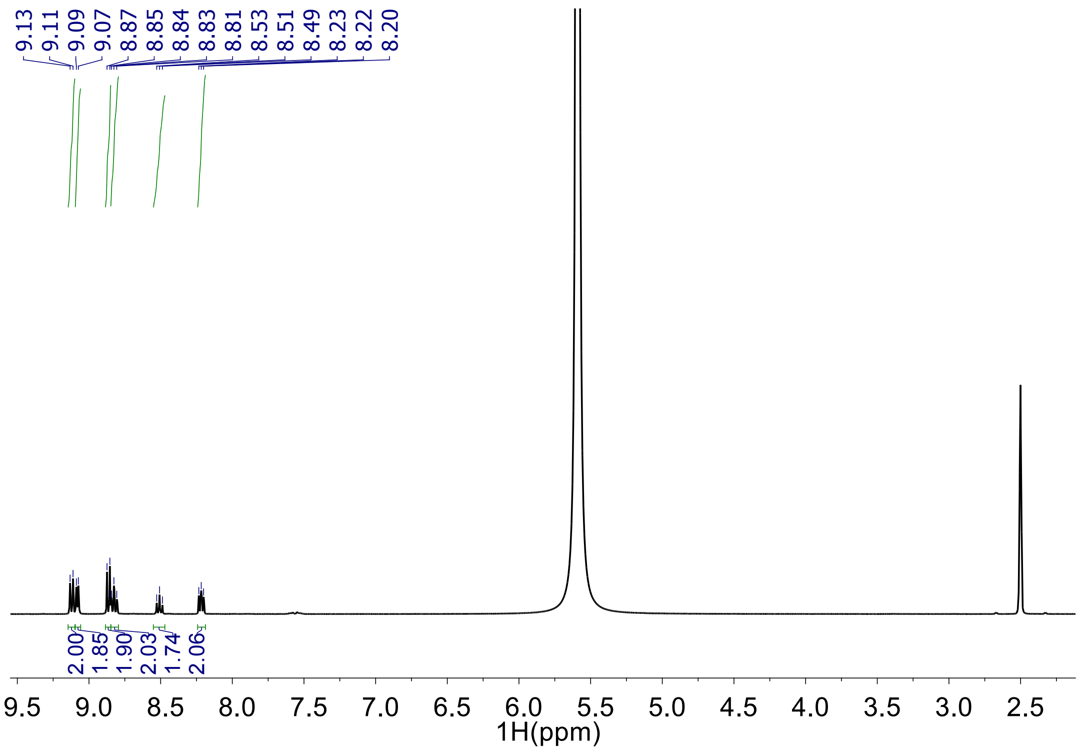

Figure S25. ^{1}H-NMR spectra (400 MHz, DMSO-D_6) of free terpyridine after the addition of 2 equiv. of trifluoroacetic acid.

Table S6. Acids screened for the protonation of complex **2** to yield **2H⁺** in DMF at 25 °C.

Acid	pK_a in H_2O	Reaction to give **2H⁺**?
HCl	-5.9	No, apparent decomplexation
p-toluensulfonic acid	-2.8	Yes
2,6-dimethylbenzoic acid	3.36	Yes
Nitrobenzoic acid	3.41	Yes
Formic acid	3.75	Yes
Toluic acids	3.91	Yes
Benzoic acid	4.19	Yes
Phenylacetic acid	4.31	Yes
Acetic acid	4.75	Yes
Pyridinium triflate	5.21	Yes
Dimethylformamidium triflate	6.1	Yes
Lutidinium perchlorate	6.72	No
Boric acid	9.2	No
Phenol	10	No
Ethanol	16	No

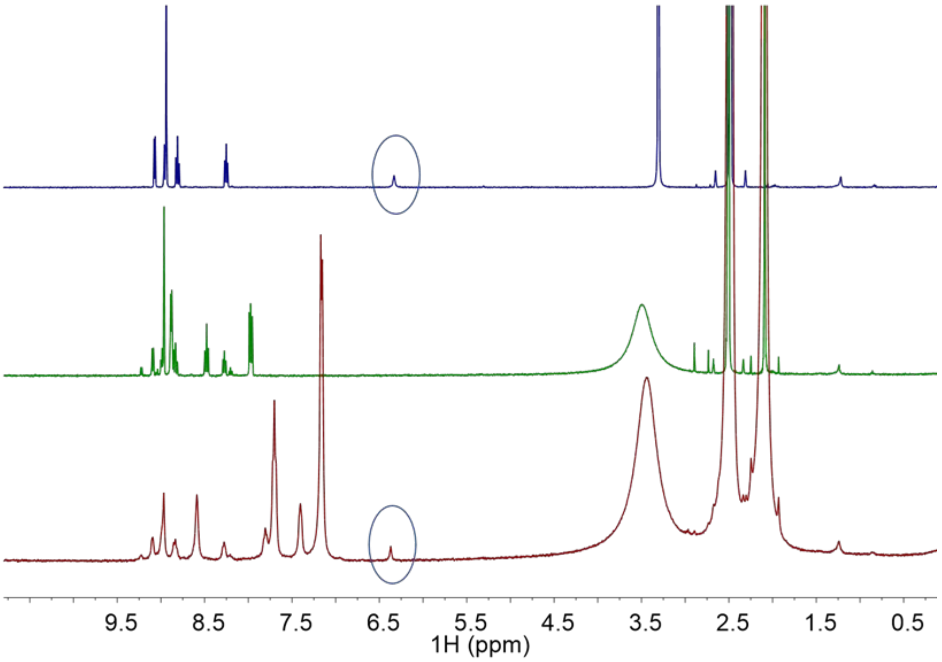

Figure S26. ^{1}H-NMR spectra (400 MHz, DMSO-D_6) of **2** (blue trace, 2.6 mM), of **2** upon addition of 5 equiv. of PyHOTf (**2H⁺**, green trace) and of **2H⁺** upon addition of 5 equiv. of 2,6-lutidine (red trace). The resonance at δ = 6.35 ppm is assigned to the proton in the hydroxide ligand.

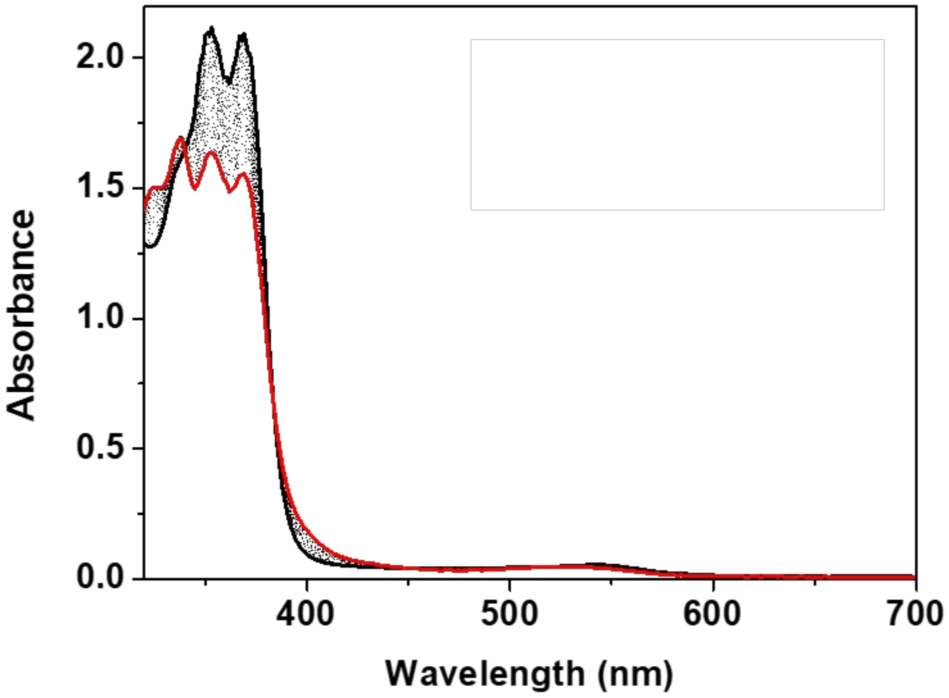

Figure S27. Change in the electronic absorption spectra of **2** upon the progressive addition of 1 equiv. of pyridinium triflate to yield **2H⁺** (red trace, 15 equiv. pyridinium triflate).

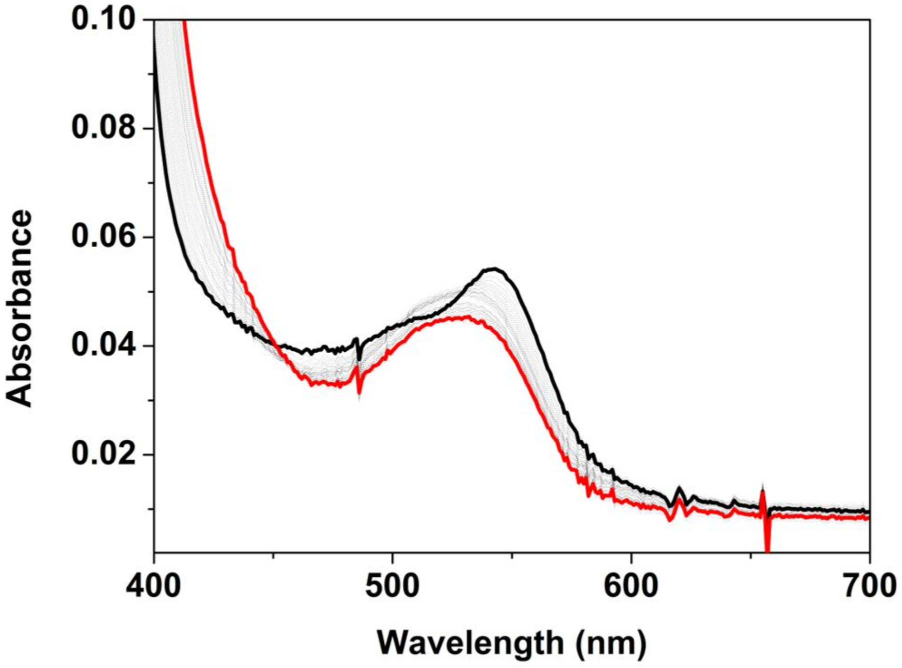

Figure S28. Change in the region between 400 and 700 nm of the electronic absorption spectra of **2** (black trace) upon the progressive addition of 1 equiv. of pyridinium triflate to yield **2H⁺** (red trace, 15 equiv. pyridinium triflate).

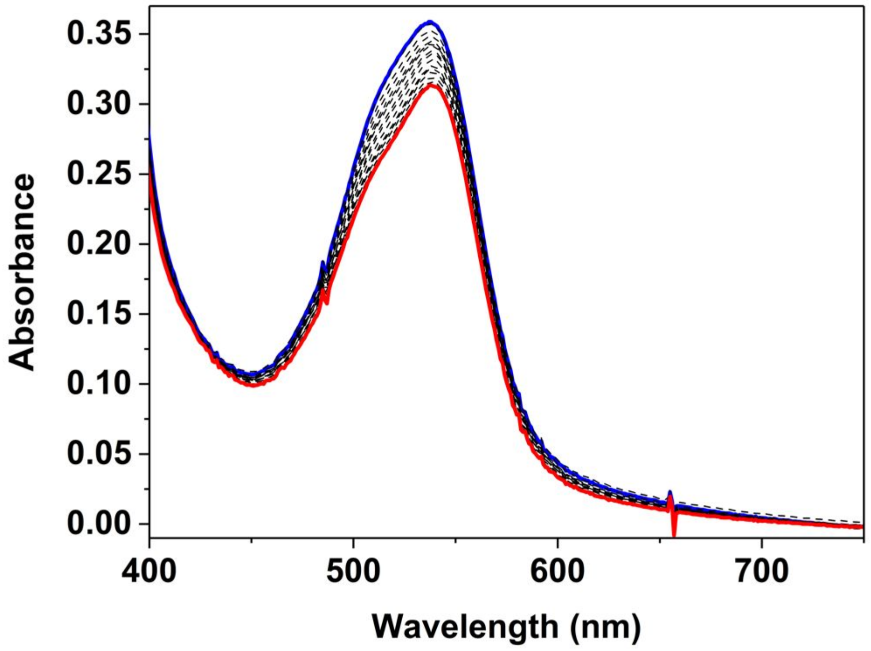

Figure S29. Change in the region between 400 and 700 nm of the electronic absorption spectra of **2H+** (blue trace) upon the progressive addition of 1 equiv. of 2,6-lutidine up to 15 equiv. (red trace). A partial restoration of the feature typical of **2** is observed in the red trace.

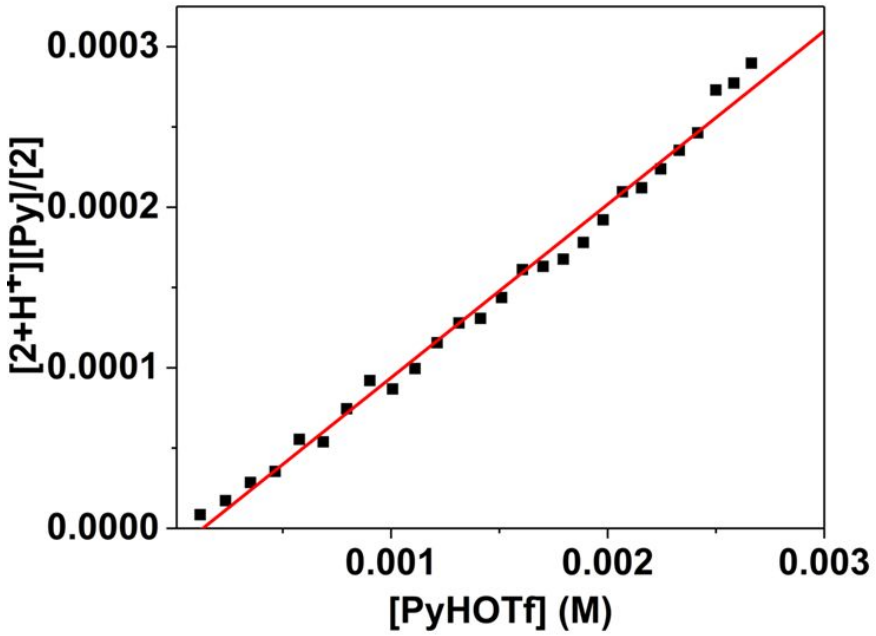

Figure S30. Plot of [PyHOTf] vs [**2H+**][Py]/[**2**]. Slope = 0.11

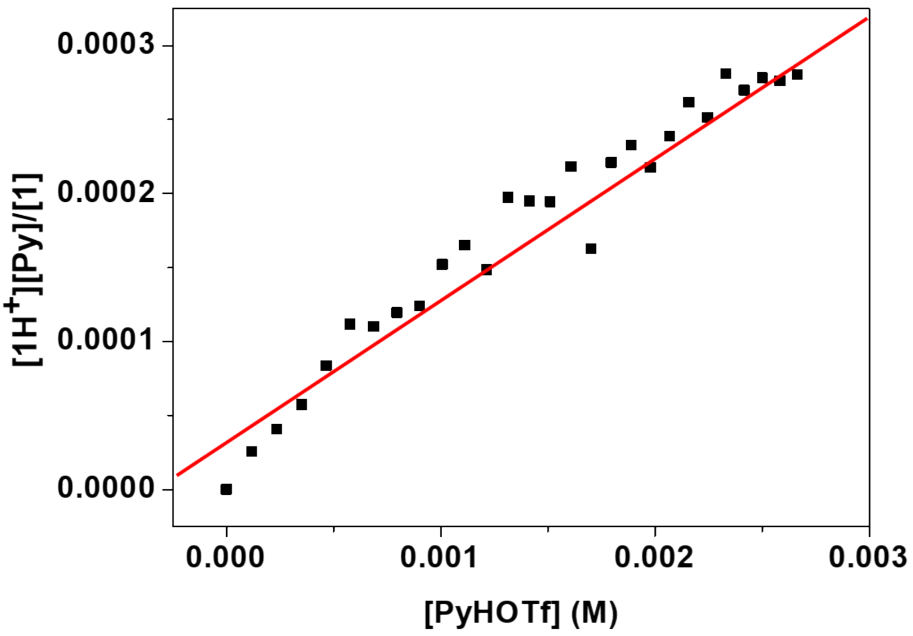

Figure S31. Plot of [PyHOTf] vs [**2H**$^+$][Py]/[**2**] considering the absorbance at λ = 545 nm. Slope = 0.10.

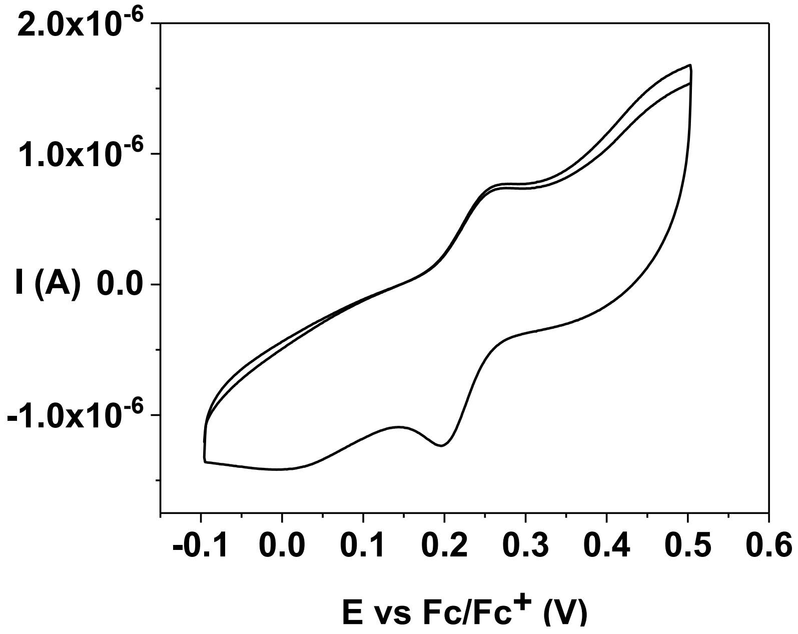

Figure S32. Cyclic voltammetry of **2H**$^+$ obtained by adding 1 equiv. of PyHOTf to **2** (DMF, 1 mM), with a focus on the redox wave at $E_{1/2}$ = 0.22 V.

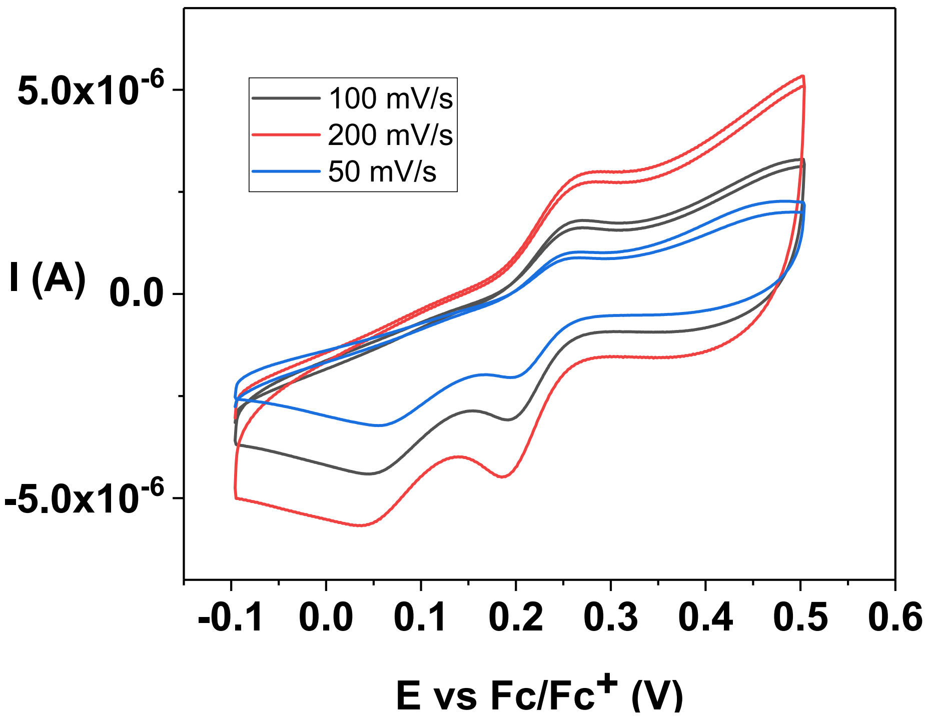

Figure S33. Cyclic voltammetry of **$2H^+$** obtained by adding 2 equiv. of PyHOTf to **2** (DMF, 1 mM), with a focus on the redox wave at $E_{1/2}$ = 0.22 V. Blue trace : scan rate 0.05 V s^{-1}, black trace: scan rate 0.1 V s^{-1}, red trace: 0.2 V s^{-1}.

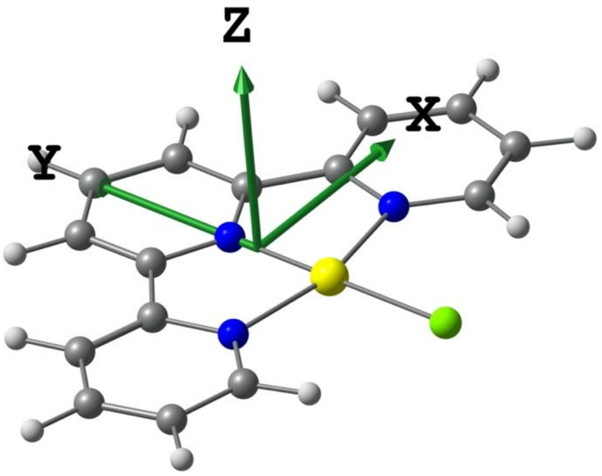

Figure S34. Optimized molecular structure of **1.**

Table S7. Atomic coordinates of optimized structure of **1**.

Au	0.000000000	0.000000000	0.000000000
Cl	0.000000000	-2.287016749	0.000000000
N	2.014413934	0.313342048	0.000000000
N	0.000059116	1.966910494	-0.000121867

N	-2.014360226	0.313346833	0.002918553
C	2.963719264	-0.632361113	0.000314639
H	2.621359501	-1.663434985	0.005904081
C	4.311739297	-0.284349468	-0.006967380
H	5.058928440	-1.073333423	-0.007150976
C	4.667370683	1.061160402	-0.013861409
H	5.714222094	1.356035059	-0.020121485
C	3.669665556	2.037016954	-0.012570356
H	3.920785270	3.094494821	-0.017576794
C	2.335957071	1.652692319	-0.005120240
C	1.201894111	2.580018732	-0.003766314
C	1.218373633	3.973951376	-0.002203227
H	2.162336559	4.511963192	-0.003153193
C	0.000075061	4.657861159	0.002330506
H	0.000087109	5.745347628	0.004181182
C	-1.218297211	3.973931738	0.004903264
H	-2.162255349	4.512008432	0.008955438
C	-1.201794091	2.580033731	0.003537988
C	-2.335889146	1.652717839	0.004356510
C	-3.669555247	2.037115349	0.006593810
H	-3.920457252	3.094691001	0.008801955
C	-4.667365487	1.061338273	0.006706519
H	-5.714178244	1.356297859	0.009104591
C	-4.311695070	-0.284170190	0.003599737
H	-5.058804357	-1.073168925	0.002314597
C	-2.963661785	-0.632329075	0.002027338
H	-2.621431546	-1.663510520	-0.001145959

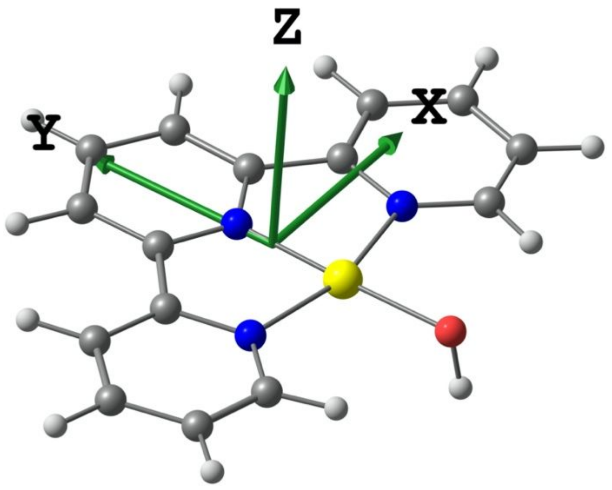

Figure S35. Optimized molecular structure of **2.**

Table S8. Atomic coordinates of optimized structure of **2**.

Au	0.000000000	0.000000000	0.000000000
O	0.000000000	-1.981090347	0.000000000
N	-2.006263957	0.307870068	0.023806548
N	0.003221354	1.962688896	0.072795245
N	2.007664126	0.302187567	0.000000000
C	-2.945292610	-0.646292743	0.011327596
C	-4.296097732	-0.308686422	0.037113276
C	-4.658769115	1.035027400	0.078052873
C	-3.668639784	2.019630095	0.095876225
C	-2.332393917	1.644701280	0.069085272
C	-1.197100153	2.577236402	0.090012757
C	-1.213296221	3.970886250	0.123757273
C	0.007536620	4.651818782	0.135419532
C	1.226152865	3.967295395	0.111096456
C	1.205449445	2.573723346	0.076819003
C	2.337765107	1.638124147	0.041175069
C	3.675135395	2.009777904	0.049519700
C	4.662421365	1.022723071	0.017230835
C	4.295794432	-0.320017968	-0.020319843
C	2.943920318	-0.654377453	-0.027548398
H	-2.584524731	-1.671857048	-0.015514246
H	-5.040103834	-1.100673472	0.025316350
H	-5.707704678	1.322150455	0.098917235
H	-3.928727510	3.074490170	0.131583973
H	-2.155388164	4.512016253	0.139179024
H	0.009252301	5.739073209	0.161177294
H	2.170068326	4.505467555	0.117786956
H	3.937900745	3.064045044	0.082691162
H	5.712263110	1.307085364	0.023866177
H	5.037718138	-1.113675100	-0.043600808
H	2.579786253	-1.678890182	-0.051649542
H	-0.032454772	-2.252823575	-0.935974169

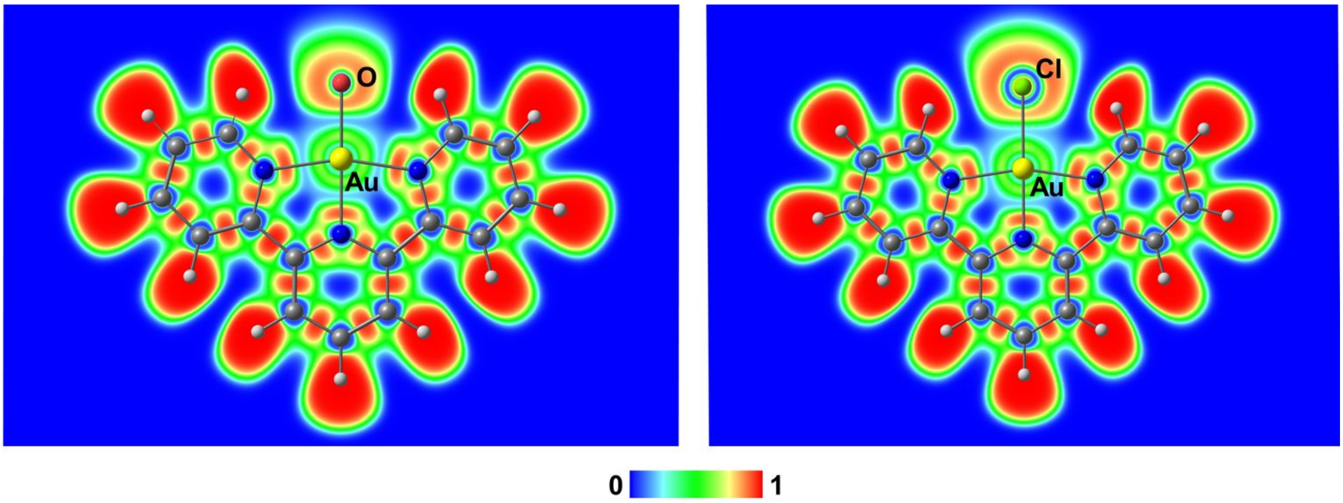

Figure S36. ELF plots for **2** (left) and **1** (right). The scale in the center refers to the probability of finding electron density along the depicted plane in the range 0-1, from the lowest (0, blue) to the highest (1, red).

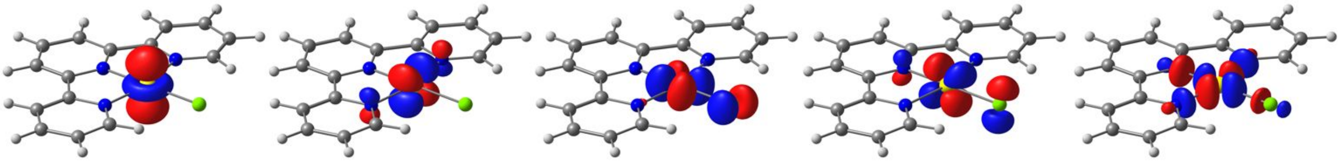

Figure S37. *AILFT* CASSCF(8,5) orbitals of **1** at 0.08 au isosurface value. The orbitals are energetically ordered from left to right (d(z^2), E=0.00 eV; d(xz), E=0.48 eV; d(xy), E=0.70 eV; d(yz), E=0.71 eV; d(x^2-y^2), E=5.61 eV).

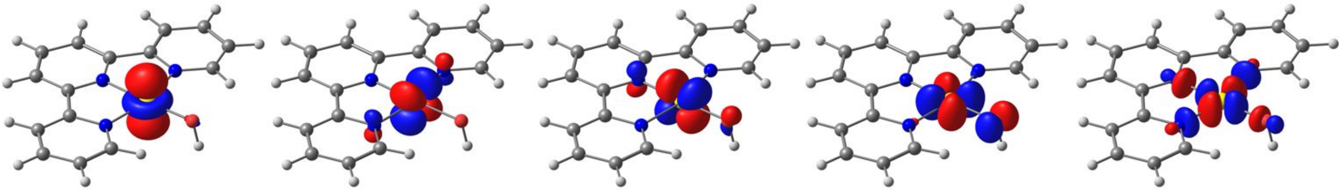

Figure S38. *AILFT* CASSCF(8,5) orbitals of **2** at 0.08 au isosurface value. The orbitals are energetically ordered from left to right (d(z^2), E=0.00 eV; d(xz), E=0.33 eV; d(yz), E=0.38 eV; d(xy), E=1.02 eV; d(x^2-y^2), E=5.98 eV).

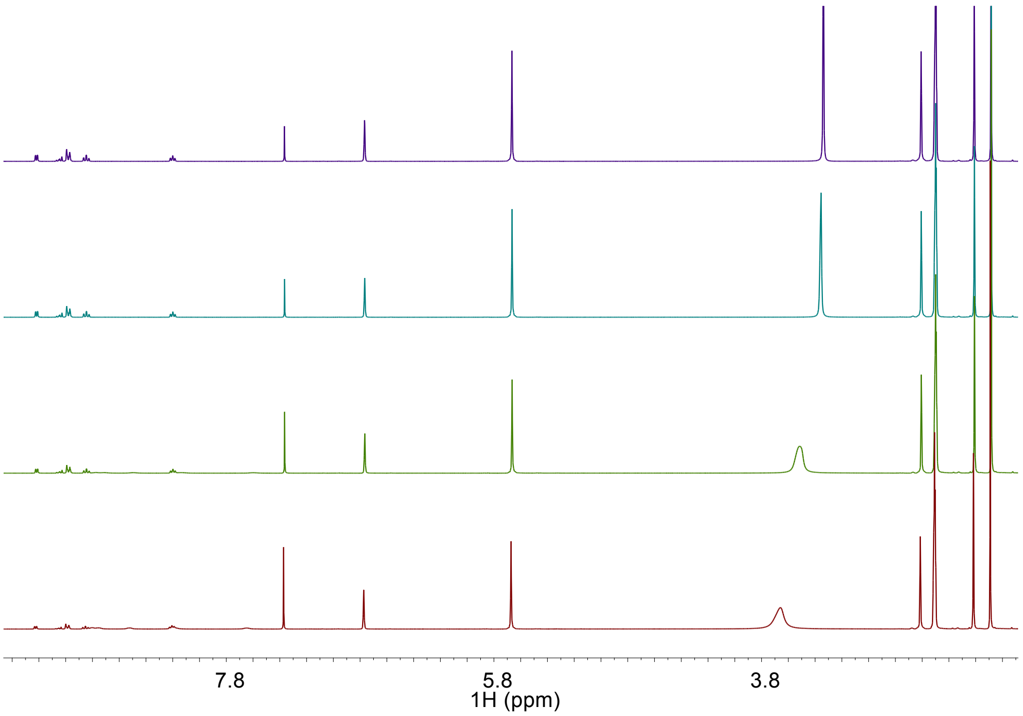

Figure S39. ^{1}H NMR spectra of the reaction between **1** and CHD (100 equiv.) in DMSO-D_6; purple = 0 s, blue = 2 h, green = 5 h, red = 24 h; benzene C–H: δ = 7.36 ppm; H_2O: δ = 3.33-3.72 ppm.

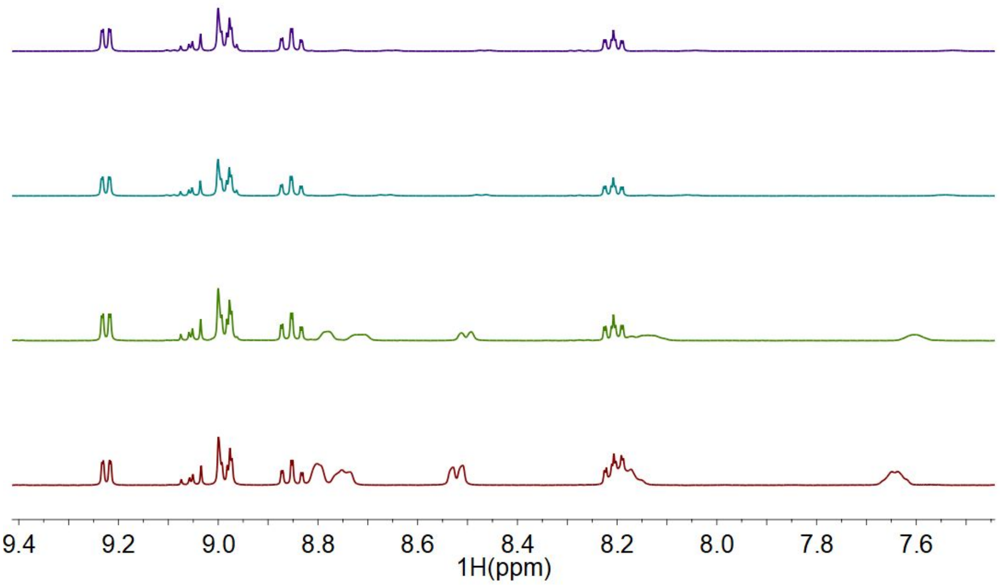

Figure S40. Zoom of the aromatic region (7.5-9.4 ppm) of the ^{1}H NMR spectra of the reaction between **1** and CHD (100 equiv.) in DMSO-D_6.

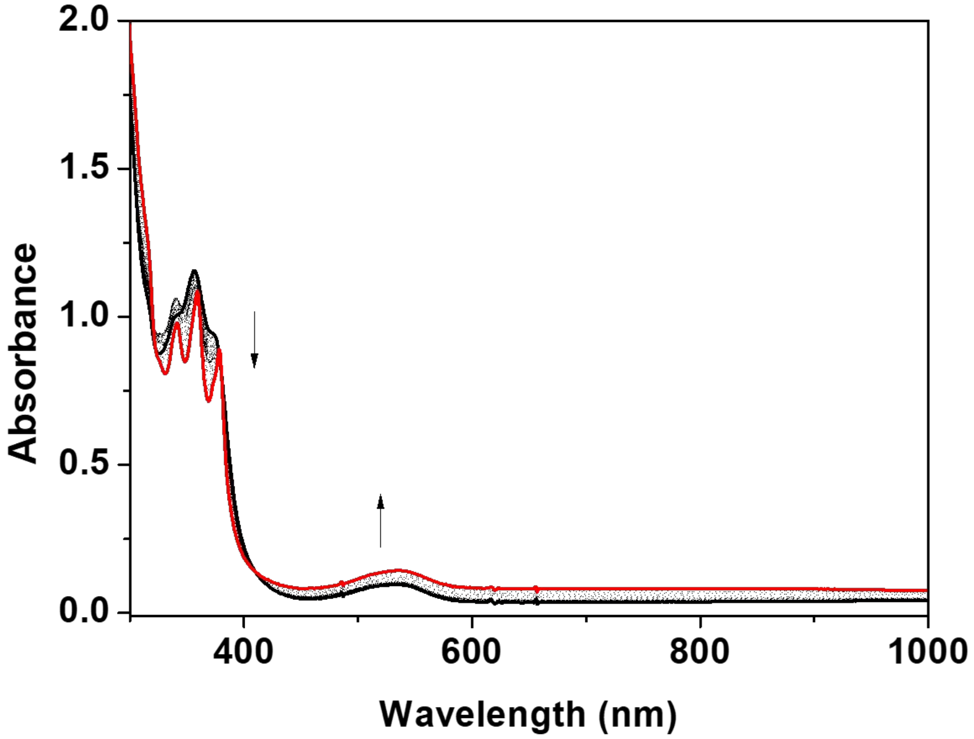

Figure S41. Electronic absorption spectra of the reaction between **1** (0.15 mM, DMF, 25 °C) and DHA (500 equiv.). Black trace = 0 s, red trace = 2500 s.

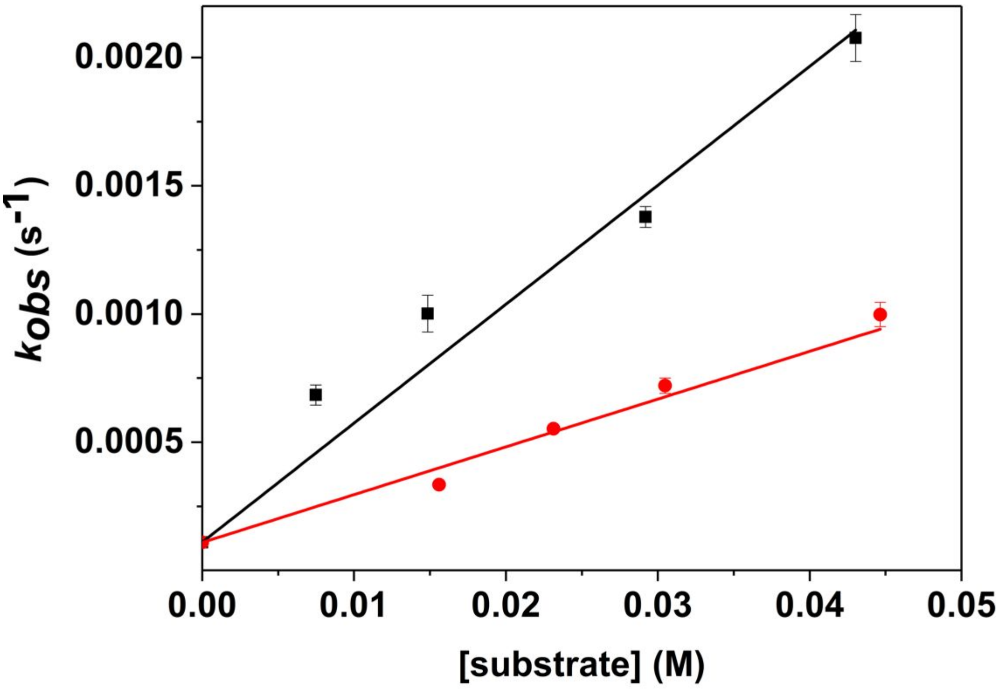

Figure S42. Plot of k_{obs} versus [substrate] determined for the reaction between complex **1** and DHA-[*H*]$_4$ (black trace) and DHA-[*D*]$_4$ (red trace).

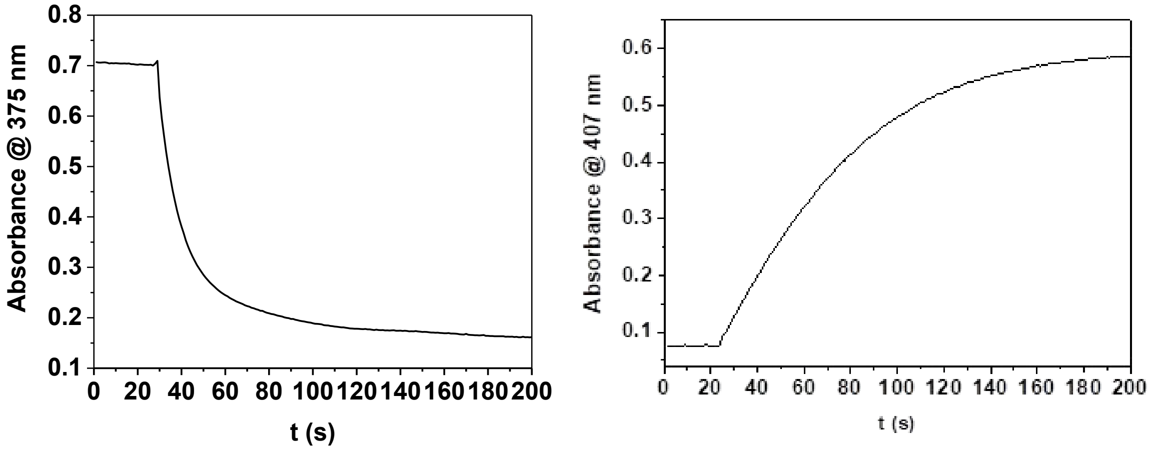

Figure S43. Absorbance data versus time for the reaction of **1** (left) and **2** (right) with 4-CH3O-2,6-DTBP.

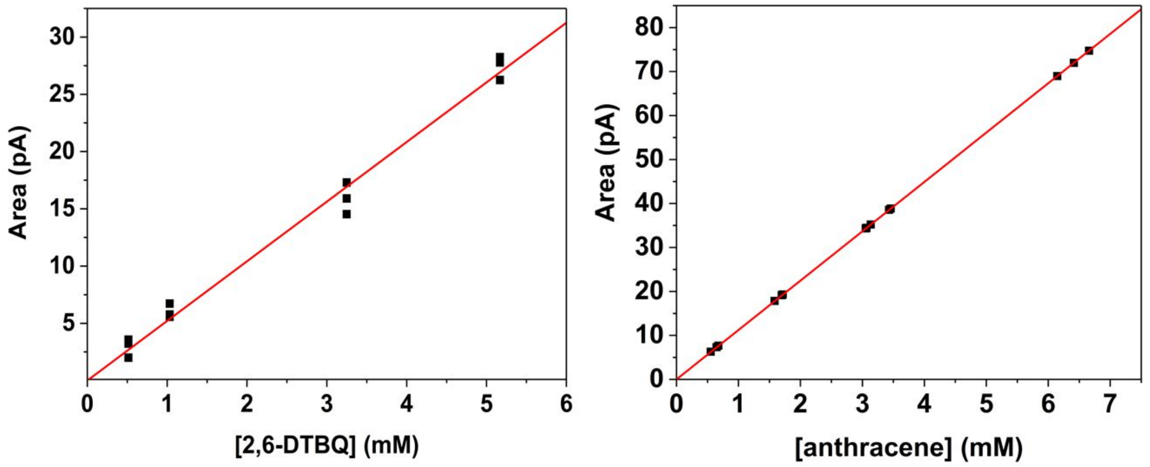

Figure S44. Calibration curves for the GC-FID determination of 2,6-DTBQ (left) and anthracene (right).

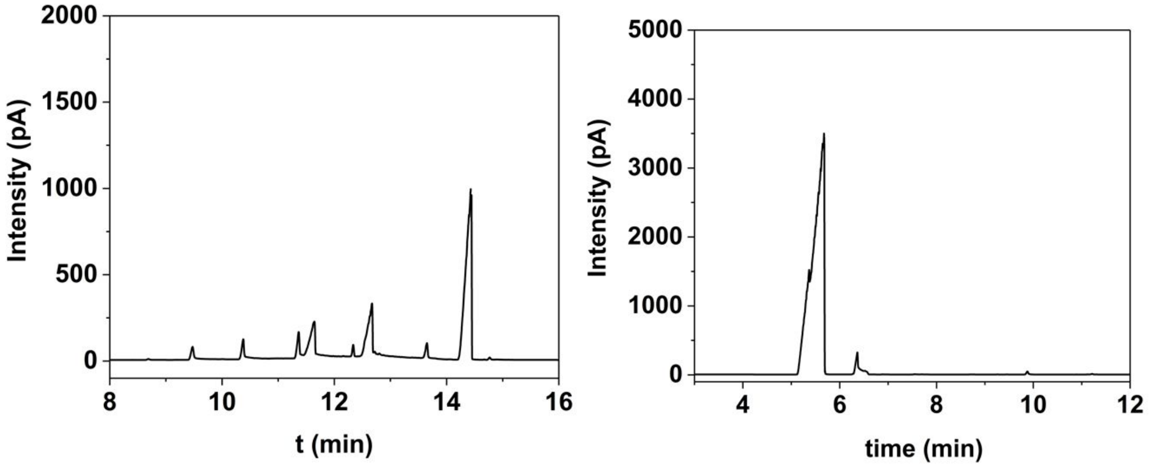

Figure S45. Chromatograms for the quantification of 2,6-DTBQ in the reaction between 50 equiv. 4-CH3O-2,6-DTBP and 1 (left) anthracene in the reaction between 1 and 150 equiv. of DHA (right).

References

1. Pitteri, B.; Marangoni, G.; Visentin, F.; Bobbo, T.; Bertolasi, V.; Gilli, P., Equilibrium and kinetic studies of (2,2′:6′,2″-terpyridine)gold(III) complexes. Preparation and crystal structure of [Au(terpy)(OH)][ClO_4]$_2$. *J. Chem. Soc., Dalton Trans.* **1999,** *5* (5), 677-682.
2. Chan, K. S.; Li, X. Z.; Dzik, W. I.; de Bruin, B., Carbon−Carbon Bond Activation of 2,2,6,6-Tetramethyl-piperidine-1-oxyl by a Rh^{II} Metalloradical: A Combined Experimental and Theoretical Study. *J. Am. Chem. Soc.* **2008,** *130* (6), 2051-2061.
3. Lee, J. Y.; Peterson, R. L.; Ohkubo, K.; Garcia-Bosch, I.; Himes, R. A.; Woertink, J.; Moore, C. D.; Solomon, E. I.; Fukuzumi, S.; Karlin, K. D., Mechanistic Insights into the Oxidation of Substituted Phenols via Hydrogen Atom Abstraction by a Cupric–Superoxo Complex. *J. Am. Chem. Soc.* **2014,** *136* (28), 9925-9937.
4. Arunkumar, C.; Lee, Y.-M.; Lee, J. Y.; Fukuzumi, S.; Nam, W., Hydrogen-Atom Abstraction Reactions by Manganese(V)– and Manganese(IV)–Oxo Porphyrin Complexes in Aqueous Solution. *Chem. Eur. J.* **2009,** *15* (43), 11482-11489.
5. APEX3 v2017.3-0, B. A. I., Madison, WI, USA.
6. SAINT v8.38A, Bruker AXS Inc., Madison, WI, USA.
7. Krause, L.; Herbst-Irmer, R.; Sheldrick, G. M.; Stalke, D., Comparison of silver and molybdenum microfocus X-ray sources for single-crystal structure determination. *J. Appl. Cryst.* **2015,** *48* (1), 3-10.
8. Sheldrick, G., SHELXT - Integrated space-group and crystal-structure determination. *Acta Cryst.* **2015,** *71* (1), 3-8.
9. Sheldrick, G., Crystal structure refinement with SHELXL. *Acta Cryst.* **2015,** *71* (1), 3-8.
10. Dolomanov, O. V.; Bourhis, L. J.; Gildea, R. J.; Howard, J. A. K.; Puschmann, H., OLEX2: a complete structure solution, refinement and analysis program. *J. Appl. Cryst.* **2009,** *42* (2), 339-341.
11. Đurović, M. D.; Bugarčić, Ž. D.; Heinemann, F. W.; van Eldik, R., Substitution versus redox reactions of gold(III) complexes with l-cysteine, l-methionine and glutathione. *Dalton Trans.* **2014,** *43* (10), 3911-3921.
12. Stoll, S.; Schweiger, A., EasySpin, a comprehensive software package for spectral simulation and analysis in EPR. *J. Mag. Res.* **2006,** *178* (1), 42-55.
13. Lu, T.; Chen, F., Multiwfn: A multifunctional wavefunction analyzer. *J. Comput. Chem.* **2012,** *33* (5), 580-592.
14. Chemcraft ver. 1.8 (build 164).
15. Wu, A.; Masland, J.; Swartz, R. D.; Kaminsky, W.; Mayer, J. M., Synthesis and Characterization of Ruthenium Bis(β-diketonato) Pyridine-Imidazole Complexes for Hydrogen Atom Transfer. *Inorg. Chem.* **2007,** *46* (26), 11190-11201.
16. Hollis, L. S.; Lippard, S. J., Aqueous chemistry of (2,2',2"-terpyridine)gold(III). Preparation and structures of chloro(2,2',2"-terpyridine)gold dichloride trihydrate ([Au(terpy)Cl]Cl_2.3H_2O) and the mixed valence gold(I)-gold(III) salt bis[chloro(2,2',2"-terpyridine)gold] tris(dichloroaurate) tetrachloroaurate ([Au(terpy)Cl]2[AuCl2]3[AuCl4]). *J. Am. Chem. Soc.* **1983,** *105* (13), 4293-4299.
17. Liu, H.-Q.; Cheung, T.-C.; Peng, S.-M.; Che, C.-M., Novel luminescent cyclometaiated and terpyridine gold(III) complexes and DNA binding studies. *J. Chem. Soc., Chem. Commun.* **1995,** (17), 1787-1788.
18. Czerwińska, K.; Golec, M.; Skonieczna, M.; Palion-Gazda, J.; Zygadło, D.; Szlapa-Kula, A.; Krompiec, S.; Machura, B.; Szurko, A., Cytotoxic gold(III) complexes incorporating a 2,2′:6′,2″-terpyridine ligand framework – the impact of the substituent in the 4′-position of a terpy ring. *Dalton Trans.* **2017,** *46* (10), 3381-3392.
19. Warren, J. J.; Tronic, T. A.; Mayer, J. M., Thermochemistry of Proton-Coupled Electron Transfer Reagents and its Implications. *Chem. Rev.* **2010,** *110* (12), 6961-7001.
20. Kundu, S.; Chernev, P.; Engelmann, X.; Chung, C. S.; Dau, H.; Bill, E.; England, J.; Nam, W.; Ray, K., A cobalt(II) iminoiodane complex and its scandium adduct: mechanistic promiscuity in hydrogen atom abstraction reactions. *Dalton Trans.* **2016,** *45* (37), 14538-14543.
21. Lovisari, M., McDonald, A. R., Hydrogen Atom Transfer Oxidation by a Gold-Hydroxide complex. *Inorg. Chem.* **2020**, *59*, 6, 3659-3665.
